# The impact of lowbush blueberry (*Vaccinium angustifolium* Ait.) and cranberry (*Vaccinium macrocarpon* Ait.) pollination on honey bee (*Apis mellifera* L.) colony health status

**DOI:** 10.1371/journal.pone.0227970

**Published:** 2020-01-24

**Authors:** Claude Dufour, Valérie Fournier, Pierre Giovenazzo

**Affiliations:** 1 Département de biologie, Université Laval, Québec, Québec, Canada; 2 Centre de recherche et innovation sur les végétaux, Université Laval, Québec, Québec, Canada; University of New England, AUSTRALIA

## Abstract

Commercial lowbush blueberry (*Vaccinium angustifolium* Ait.) and cranberry (*Vaccinium macrocarpon* Ait.) crops benefit from the presence of honey bee (*Apis mellifera* L.) for pollination. Unfortunately, beekeepers are observing negative impacts of pollination services on honey bee colonies. In this study, we investigated three beekeeping management strategies (MS) and measured their impact on honey bee colony health and development. Experimental groups (five colonies/MS) were: A) Control farmland honey producing MS (control MS); B) Blueberry pollination MS (blueberry MS); C) Cranberry pollination MS (cranberry MS) and D) Double pollination MS, blueberry followed by cranberry (double MS). Our goals were to 1) compare floral abundance and attractiveness of foraging areas to honey bees between apiaries using a Geographic Information System, and 2) compare honey bee colony health status and population development between MS during a complete beekeeping season. Our results show significantly lower floral abundance and honey bee attractiveness of foraging areas during cranberry pollination compared to the other environments. The blueberry pollination site seemed to significantly reduce brood population in the colonies who provided those services (blueberry MS and double MS). The cranberry pollination site seemed to significantly reduce colony weight gain (cranberry MS and double MS) and induce a significantly higher winter mortality rate (cranberry MS). We also measured significantly higher levels of Black queen cell virus and Sacbrood virus in the MS providing cranberry pollination (cranberry MS and double MS).

## Introduction

Landscapes have been altered by the increasing presence of intensively managed monocultures. One of the most concerning results of these changes is a decrease in the quantity, quality and diversity of floral resources available to honey bees, which in turn has led to a decrease in honey bee productivity [1–3].

Commercial pollination by honey bees plays a major role in agriculture and sustains the yield and quality of many agricultural crops around the world [4]. Lowbush blueberries (*Vaccinium angustifolium* Ait.) and cranberries (*Vaccinium macrocarpon* Ait.) are important berry crops in Canada that benefit from the presence of the non-native honey bee as a pollinator to ensure optimal fruit set and profitability [5, 6]. When honey bees are present as pollinators, lowbush blueberry fruit set increases up to 70% to almost 100% [7], and up to 50% for cranberry [8, 9].

**Fig 1:**
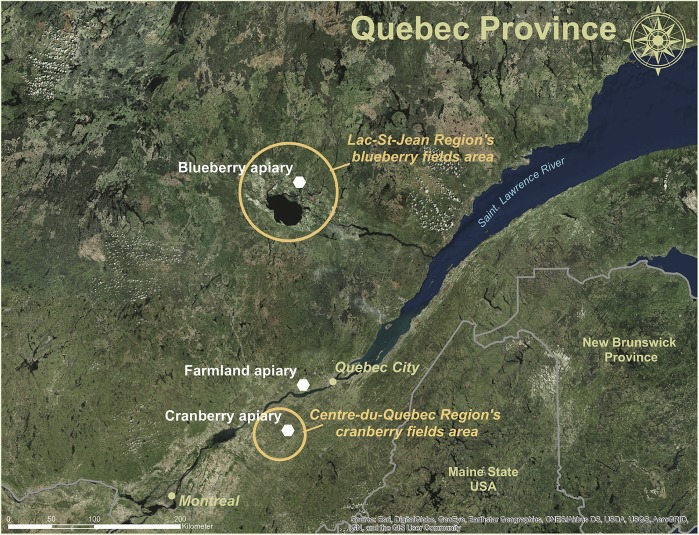
Location of the study apiaries. Location of the farmland, blueberry field and cranberry field apiaries, against the location of the most important regions for blueberry and cranberry production in the province of Quebec, Canada. Sources: Esri, DigitalGlobe, GeoEye, i-cubed, USDA FSA, USGS, AEX, Getmapping, Aerogrid, IGN, IGP, swisstopo, and the GIS User Community. Maps were created using ArcGIS® software by Esri. ArcGIS® and ArcMap^™^ are the intellectual property of Esri and are used herein under license. Copyright Esri. All rights reserved. For more information about Esri® software, please visit www.esri.com.

In the province of Quebec, Canada, lowbush blueberry crops cover 29800 hectares, and cranberry crops 4900 hectares [10]. Quebec’s cranberry industry is the second largest worldwide, after that of Wisconsin (USA) [11]. Most of Quebec’s blueberry fields (75%) are located in the Lac-St-Jean region [12], and the great majority of its cranberry farms (90%) in the Centre-du-Quebec region [11, 13] (Fig 1). Renting colonies for pollination services is a significant source of income for commercial beekeepers, representing 27% of the total revenues from all beekeeping activities in the province [14].

Unfortunately, although migratory beekeeping contributes to the pollination needs of many cropping systems, providing pollination services can have negative impacts on honey bee colonies due to stress from being transported from site to site [15–18] and poor nutrition due to lower flower diversity [19]. Honey bees are generalist pollinators that feed on diversified plant species [20]. Rich ecosystems with abundant resources contribute to the pollinator health [21]. Monofloral pollen diets might not provide sufficient nutrients to ensure good honey bee health [21–23]. Thus, bees benefit on foraging on a wide variety of flower species to satisfy a more balanced diet [24]. Lowbush blueberry is known for either its good pollen production or seed production, but not both [25], and low protein content in pollen [26]. It also has a low nectar production [27, 28]. Cranberry flowers produce an abundance of pollen [29] but low quantities of nectar [29, 30]. Cranberry pollen protein appears in higher concentration than blueberry pollen [31]. The protein content in pollen is necessary for honey bee larval development [32, 33]. A pollen shortage during a worker’s larval development will shorten her lifespan, impair her ability to communicate with sisters, decrease her homing ability and induce poor vigor and other physical limitations [34]. Carbohydrates from nectar and honey are the essential energy sources for honey bees [35].

Large croplands such as lowbush blueberry and cranberry fields often lack flower diversity [36, 37], which may reduce sources of nutrition for honey bees and contribute to harming their development [38]. For instance, Bizotto *et al*. [39] measured a significant reduction of brood production caused by pollen and nectar shortage during provision of apple pollination services. During commercial pollination, honey bee colonies are also exposed to a high risk of viral infections caused by high colony densities [40] and reduced lifespan because of oxidative stress caused by transhumance [16].

In this study, we followed honey bee colonies in four different pollination management strategies (MS): honey producing vs blueberry pollination and / or cranberry pollination. Our goals were to 1) compare the honey bee foraging potential of the landscape surrounding the apiaries using a Geographic Information System, and 2) compare honey bee colony health status and population development among MS during a complete beekeeping season. We predicted that lower floral diversity from pollination crops would have a negative impact on the honey bee colony development and anticipated a higher prevalence of parasites and pathogens in colonies providing pollination services.

## Material and methods

### Experimental treatments and colony management

No permits were required for this research. Colonies were owned by our research center (Centre de recherche en sciences animales de Deschambault-CRSAD, Quebec, Canada) and verbal agreements were taken with the landowners of the various apiaries.

Twenty colonies of similar strength (total brood area) were selected from among the livestock at our bee research facility CRSAD. Young sister queens had been introduced in these colonies in July 2015. These queens were hybrid Italian selected stock from our honey bee breeding program. Colonies were randomly assigned to the following different management strategies (MS) (5 colonies/MS): A) control MS; B) blueberry MS; C) cranberry MS and D) double MS.

Field work was conducted between May 2016 and May 2017 at three different apiary sites in the province of Quebec: a diversified farmland area (composed of different small crops mixed with natural and free growing lands) that served as the farmland apiary in Pont-Rouge (71° 39' 49.966'' N; 46° 45' 25.129'' O), a commercial lowbush blueberry field in St-Ludger-de-Milot in the Lac-St-Jean region (71° 50' 6.462'' N; 48° 52' 42.292'' O) (from June 7, 2016 to June 23, 2016) and a commercial cranberry field in Notre-Dame-de-Lourdes in the Centre-du-Quebec region (71° 52' 19.753'' N; 46° 16' 37.274'' O) (from June 24, 2016 to July 19, 2016). Location of apiaries was selected to represent the standard agricultural profile of regional farmland, blueberry and cranberry fields (Fig 1).

At the beginning of the study, in May 2016, all colonies started at the farmland apiary (20 colonies). One group of five colonies remained at this site for the entire study (control MS; n = 5 colonies) while 15 of them were transported following the dates presented in Table 1 to provide pollination services for blueberries (blueberry MS; n = 5 colonies), cranberries (cranberry MS; n = 5 colonies) or both (double MS; n = 5 colonies) according to the management strategy schedule (Table 1). Colonies were moved at night (between 23h00 and 05h00) on an open truck. From May 20 to August 25, 2016, all colonies were comprised of two brood chambers, while honey supers were added when needed. At the beginning of September, honey supers were removed, and colonies were reduced to one brood chamber. Fall feeding started in mid-September 2016 and all colonies were given 24 liters of a sucrose 2:1 solution using a top box feeder (Wooden Miller feeder # FE-1100 at Propolis-etc, Beloeil, Quebec). Colonies received a Thymovar® anti-*Varroa* treatment starting on September 12, 2016, followed by an oxalic acid treatment on November 5, 2016 (drip method: 35 g/L in a sucrose 1:1 solution, 5 mL between frames of the hive body crowded with honey bees). Colonies were wintered indoors in an environmentally controlled room (4–5°C, 50–60% RH) from November 14, 2016 to April 19, 2017 and then moved in a spring apiary in Deschambault, Quebec near the bee research facility until May 2017.

**Table 1:** Colony management strategy schedule.

	Location (apiary) per pollination period			
Management strategies (MS)	No pollination services (May 2016 to June 6, 2016)	Blueberry pollination (June 7, 2016 to June 23, 2016)	Cranberry pollination (June 24, 2016 to July 19, 2016)	No pollination services (July 20, 2016 to May 2017)
**A) Control MS (n = 5 colonies)** (farmland honey-producing)	FARMLAND			
**B) Blueberry MS (n = 5 colonies)** (pollination services in blueberry field)	FARMLAND	BLUEBERRY	FARMLAND	
**C) Cranberry MS (n = 5 colonies)** (pollination services in cranberry field)	FARMLAND		CRANBERRY	FARMLAND
**D) Double MS (n = 5 colonies)** (pollination services in blueberry field followed by cranberry field)	FARMLAND	BUEBERRY	CRANBERRY	FARMLAND

Colony management strategy (MS) schedule (n = five colonies per MS). Pollination MS colonies that provided pollination services (blueberry MS, cranberry MS and double MS) were moved from one apiary to another following this schedule. When colonies were not providing pollination services, they were located at the farmland apiary.

### Landscape analysis

To assess the impact of environment on the colonies, the landscape around the apiaries was characterized within three distances: A-Proximal 1.5-km radius, B-Medial 3-km radius and C- Distal 5-km radius. Data were extracted from databases at the Ministère de l’Énergie et des Ressources naturelles du Québec and the Financière Agricole du Québec and was analysed with a GIS, ArcGIS 10.4 [41]. The first holds databases on forestlands and the latter on commercial crops. Polliniferous and melliferous potential of vegetation was characterized based on Moisan-De Serres *et al*. [42] and flowering period. The following were considered of “low foraging potential”: coniferous forests, mixed and deciduous forests (after springtime when most tree flowers have bloomed), non-melliferous and non-polliniferous crops (potatoes, most cereals, etc.), urban and artificial areas (roads, gravel pits, etc.), wetlands (after Ericaceae blooming) and water; while the following were considered of “good foraging potential”: mixed and deciduous forests (during springtime when tree flowers bloom), melliferous and polliniferous crops (fruits, grass, soya, etc.), recent clearcuts, wetlands (during Ericaceae bloom), pastures and alders (during springtime when flowers bloom).

### Precipitation

Precipitation (rain) data were extracted from the Environment Canada database at stations located within 25 km distance from the study sites (Deschambault weather station for the farmland control site (25 km), Laurierville weather station for the cranberry pollination site (18 km) and Peribonka weather station for the blueberry pollination site (21 km)).

### Colony health status

#### Colony performance

Colony weight: Hives were weighed individually throughout the season each time a honey super was added or removed. Weighing was accomplished by placing the entire colony (brood chamber and honey supers) on a platform scale (total capacity of 500 kg, minimum weight sensitivity of 0.1 kg). Data are presented as weight gain (in kilograms) throughout the season.

Honey bee brood population: The area occupied by immature worker honey bees (eggs + larvae + capped brood) in colonies was evaluated by measuring width and length of the brood surface area on each side of every brood frame. The rectangular surface obtained was multiplied by 0.8 to compensate for the elliptic form of the brood pattern. These values were added in order to calculate the total brood surface in each colony. A factor of 25 worker cells per 6.25 cm^2^ (i.e., a square inch) was used to convert the area to obtain the number of immature worker honey bees [43, 44].

#### Pathogens and parasites

**Fig 2:**
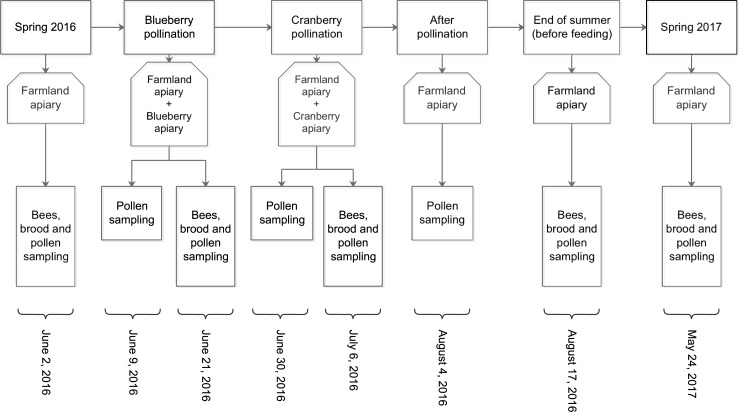
Data sampling schedule. Data sampling schedule from spring 2016 to spring 2017.

Pathogen and parasite occurrence were measured in worker honey bee larvae and nurse and forager bees. The samplings were performed according to the schedule in Fig 2. On site, each sample was rapidly placed in a container, then in a propane freezer (-20°C) packed with dry ice, for immediate freezing. Samples were then brought to the lab and stocked in a freezer at -86°C (Thermofisher -86°C FORMA 908).

Virology: Samples consisted of ten honey bee larvae (6 to 8 days old), approximately 200 nurse bees caught on brood frames and approximately 70 forager bees captured with a small vacuum at the entrance of the hive. Analyses were performed by the Animal Health Laboratory (AHL), at Guelph University in Guelph, Ontario. Honey bee larvae, nurse bees and forager bees were shipped frozen on dry ice for delivery within 24 hours. Quantification of the following viruses was assessed: Acute bee paralysis virus (ABPV), Black queen cell virus (BQCV), Chronic bee paralysis virus (CBPV), Deformed wing virus (DWV), Israeli acute paralysis virus (IAPV), Kashmir bee virus (KBV) and Sacbrood virus (SBV). Analyses on each of the three life stages (larvae, nurse and foragers bees) were performed by pooling 10 bees or larvae from each sample. The ten individual bees or larvae were selected by looking for the most intact specimens within the sample container. For adult bees, any bees that showed signs of dying at ambient temperature (i.e. 'K-flex' wings) were excluded. The pools were homogenized in 5 mL of TriReagent using a SPEX Mini-G tissue homogenizer set at 1500 oscillations per minute and run for five minutes, and were then centrifuged for 2 minutes at 1500 rpm to pellet debris before the supernatant was collected for extraction. Then, 100 μL of the homogenate were used to extract RNA with a MagMax instrument using a MagMax^™^ Pathogen RNA/DNA Kit (LifeTechnologies) and following the Whole Blood protocol from the manufacturer with the exception that 100 μL of elution buffer was used. Then, 1 μL of the homogenate were used as the template for reverse transcription (RT) using SuperScriptIII 1st Strand qPCR Supermix (ThermoFisher) in a final volume of 20 μL following the manufacturer’s instructions. From the RT products, 2 μL were used for qPCR using LightCycler 480 SYBR Green 1 Master (Roche) in a real-time instrument (LC480, Roche). The PCR conditions and primer sequences were described previously [45, 46]; AHL unpublished data). Per-bee copy numbers were obtained by multiplying the per-reaction copy number by a factor of 5,000. As 100 μL of homogenate (0.2 bee) were extracted and eluted in the same volume, 1 μL was taken to use as RT template (1/100 x 0.2 bee), resulting in a 20 μL final volume of cDNA, of which 2 μL was used as qPCR template (1/100 x 1/10 x 0.2 bee). We used “virus prevalence” to indicate the presence or absence of a given virus in each colony and we used “virus virulence” to assess the number of copies of virus per bee detected in average in each colony.

Nosemosis: Samples consisted of approximately 300 forager bees that were shaken off side frames (no brood) over a funnel mounted on a container and frozen immediately (see description above). Analyses were performed by the National Bee Diagnostic Center at Grande Prairie Regional College in Beaverlodge, Alberta. Sixty bees per sample were homogenized in 60 ml of 70% ethanol (1 ml/bee) in a Stomacher for 60 seconds at normal speed. One thousand μL of the homogenate was transferred into a small tube for further microscopic examination. The microscopic examination was conducted using a Helber bacteria counting chamber under 400x magnification with a volume of 6 μL aliquot from the tube previously prepared (pers. comm.). The Nosema spores were identified and counted with differentiating the species *N*. *ceranae* and *N*. *apis* [47]. Only *N*. *ceranae* is discussed in this paper and results are presented in number of spores per bee in average for each colony.

*Varroa destructor*: For the first *Varroa* monitoring sampling, *Varroa* mite population was estimated with the alcohol washing method on June 1^st^, 2016. Samples of 60 to 100 bees were agitated for 60 seconds in a bee shaker with alcohol (Bee shaker # BS-1001 at Propolis-etc), mounted with a mesh to separate the mites from the bees. All mites and bees were counted [48]. Results are presented in number of mites per 100 bees. For the following *Varroa* monitoring samplings, fallen *Varroa* mites were counted on sticky boards placed on the bottom boards of all hives on October 27^th^ and December 6^th^, 2016 and June 1^st^, 2017. Sticky boards covered the entire bottom surface of hives and were left in place for seven consecutive days. Results are presented in number of fallen *Varroa* per day.

#### Queen loss

All queens were identified and painted prior to the study. At each queen inspection (minimum every 10 days), we conducted a queen assessment by localizing it, evaluating her laying behavior and inspecting for physical defects. All royal cells seen were destroyed to prevent swarming.

#### Winter mortality

Colony winter mortality (2016–2017) was assessed by counting the number of colonies that survive the wintering and early spring at least until May 2017.

### Statistical analysis

Statistical analyses were performed to verify whether the blueberry pollination and/or the cranberry pollination have deleterious impacts on honey bee colony health and development compared to colonies used for honey producing only. We built this experimental model considering the honey bee colonies as experimental units [49, 50]. This assumption is based on the following knowledge of bee colony foraging behaviour: bees from each colony have a foraging environment beyond each apiary (up to 5 km radius [51–54]) and the bees from each colony within an apiary will visit different plants [1, 22]. Furthermore, experimental colonies were moved between various pollination environments (farmland (no commercial pollination services)/blueberry field/cranberry field). They were not stationary units. Because colonies were not moved in different blueberry or cranberry fields, we expect variability between colonies to be smaller than the one that could be observed when colonies are moved to different fields, and this leads to more liberal tests. To account for this kind of pseudoreplication, we fixed the significance level at α = 0.01, instead of 0.05, to reinforce the findings of our research even though we know that the inference is valid only for the selected blueberry and cranberry fields. However, we caution interpretation of significance in our results because of the small sample size and presence of pseudoreplication.

Data were analyzed using a two-way Anova model with repeated measurements. In this model, the treatment is the between-subject factor, while the time is the within-subject factor. Interaction between treatment and time is also considered in this model in order to compare the treatment profiles over time. The treatment structure involves four qualitative treatments with 5 colonies in each one. Colonies are thus nested within treatment and this factor is considered as random in the model. The treatments are the following: A) Control MS (located in the farmland apiary for the whole study); B) blueberry MS (located in the farmland apiary before and after the blueberry pollination and in the blueberry apiary during the blueberry pollination); C) cranberry MS (located in the farmland apiary before and after the cranberry pollination and in the cranberry apiary during the cranberry pollination) and D) double MS (located in the farmland apiary before and after both the blueberry and cranberry pollination; in the blueberry apiary during the blueberry pollination; and in the cranberry apiary during the cranberry pollination). Since measurements are taken on the same colonies over time, there is a correlation between those measurements. The structure of correlation that best fit the data were chosen based on the AIC criterion. Tests for normality and homogeneity of variance were carried out on the residuals of each model. Because the number of virus copies did not have a normal distribution, we used the Box-Cox approach and selected the log transformation for analysis. Following a significant effect in the model, post hoc Tukey’s tests (HSD) were performed for comparing differences between treatments and time. Means are reported ± standard error. Finally, all analyses were performed using SAS (SAS institute inc., NC, version 9.4) [55] through the Mixed procedure at the 0.01 level of significance.

## Results

### Landscape analysis

**Fig 3:**
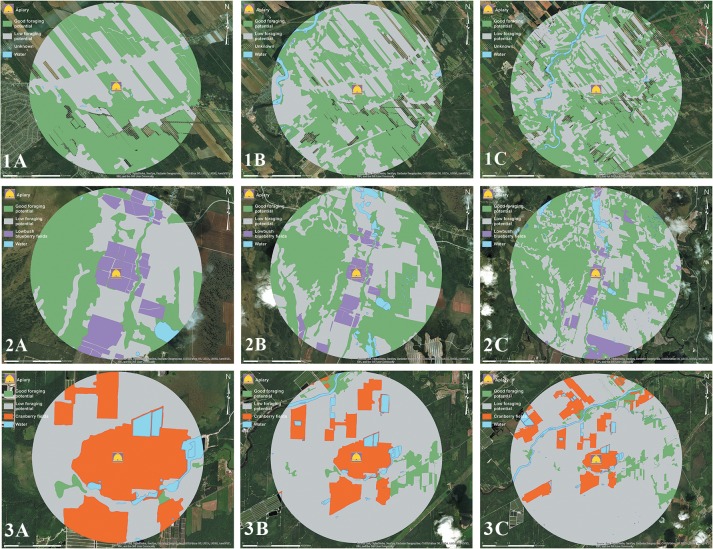
Landscape structure. Landscape structure (forestlands and farmlands) surrounding the various apiaries: 1) farmland apiary, 2) blueberry apiary and 3) cranberry apiary in a proximal 1.5-km radius (A), medial 3-km radius (B) and distal 5-km radius (C). Areas in green are those with good foraging potential for honey bees, while areas in grey are those with low foraging potential. Lowbush blueberry fields are in purple and cranberry fields are in red. Data were extracted from the Ministère de l’Énergie et des Ressources naturelles du Québec database (Provincial 4^th^ 10-year program) and the Financière Agricole du Québec database (Info-Sols 2016). Sources: Esri, DigitalGlobe, GeoEye, i-cubed, USDA FSA, USGS, AEX, Getmapping, Aerogrid, IGN, IGP, swisstopo, and the GIS User Community. Maps were created using ArcGIS® software by Esri. ArcGIS® and ArcMap^™^ are the intellectual property of Esri and are used herein under license. Copyright Esri. All rights reserved. For more information about Esri® software, please visit www.esri.com.

The farmland and blueberry apiaries presented similar percentages of good foraging potential landscape for the three radii (between 43% and 56%) (Fig 3 and Table 2). The cranberry apiary had a lower percentage of good foraging potential landscape for all foraging radii (below 10%). The following is a description of the different land types of the honey bee foraging 5K radius for each apiary (a comprehensive list of the different land types of the three honey bee foraging radii is available) (S1 Table): The good foraging potential landscape was composed mostly of mixed and deciduous forests (41%) as well as crops (8%) in the farmland apiary; wetlands (29%) and mixed and deciduous forests (19%) in the blueberry apiary; and crops (4%) and pastures (2%) for the cranberry apiary. The low foraging potential landscape was composed mostly of coniferous forests (14%), urban areas (12%), and non-melliferous/polliniferous crops (11%) in the farmland apiary; coniferous forests (24%), non-melliferous/polliniferous crops (10%) in the blueberry apiary; and forests (62%), wetlands (7%) and urban areas (3%) in the cranberry apiary. The percentage of the landscape occupied by the pollinated crop was greater for the cranberry apiary, with 46% at the 1.5-km radius compared to the blueberry apiary, with 18%.

**Table 2:** Foraging potential.

	Foraging potential of the landscape surrounding the apiaries								
Apiaries	A-Proximal 1.5-km radius			B-Medial 3-km radius			C-Distal 5-km radius		
	Good potential	Low potential	Pollinated crop	Good potential	Low potential	Pollinated crop	Good potential	Low potential	Pollinated crop
**Farmland**	56%	44%		49%	51%		52%	48%	
**Blueberry**	43%	38%	18%	51%	42%	7%	55%	39%	6%
**Cranberry**	5%	49%	46%	9%	73%	18%	9%	77%	13%

Foraging potential area (%) surrounding the apiaries according to 1.5-km, 3-km and 5-km radii based on the analysis of the landscape (forestlands and croplands).

### Precipitation

During blueberry pollination services (June 7 to June 23, 2016), rain occurred during 11 days at the blueberry site among which 6 days received more than 5 mm of rain, as opposed to one day (> 5 mm of rain) at the farmland site. During cranberry pollination (June 24 to July 19, 2016), rain occurred during 13 days at the cranberry site among which 6 days received more than 5 mm of rain, as opposed to 5 days of (four of more than 5 mm of rain) at the farmland site.

### Colony health status

#### Colony performance

Colony weight gain: Colony weight gain was similar between all groups until the cranberry pollination period (June 30, 2016, Fig 4). Average colony weight gain on July 6, 2016 was significantly lower (F_3,166_ = 6.38, p<0.001) in the cranberry MS and double MS compared to control MS and blueberry MS. This significant difference persisted (“carry-over effect”) after the cranberry pollination period and when all pollinating colonies were brought back to the farmland apiary July 21–26, August 4-15-23-30 and September 6 (respectively: F_3,166_ = 18.73; F_3,166_ = 43.98; F_3,166_ = 37.39; F_3,166_ = 44.57; F_3,166_ = 55.99, p<0.001; F_3,166_ = 63.19; F_3,166_ = 65.28; p<0.001 for all). One month after the cranberry pollination services period (August 23 to September 6), colonies in the cranberry MS presented a significantly lower weight gain than those in the double MS, although the colonies were similar until then (F_3,166_ = 55.99, p<0.001 (August 23, 2016); F_3,166_ = 63.19, p<0.001 (August 30, 2016); F_3,166_ = 65.28, p<0.001 (September 6, 2016)).

**Fig 4:**
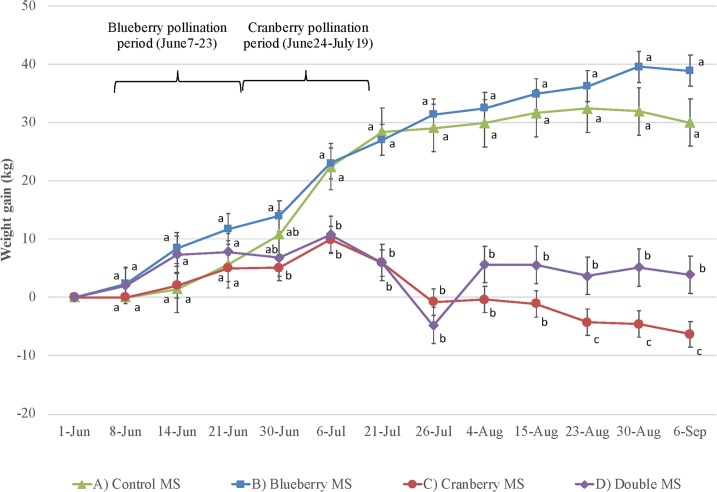
Hive weight gain. Hive weight gain (mean weight in kilograms ± SE) per treatment (n = 5 colonies per treatment) over a season (from June 1, 2016 to September 6, 2016), including surveys before pollination, during blueberry pollination and cranberry pollination and after pollination. Significant differences between treatments (p < 0.01 based on post hoc Tukey’s test—HSD) are shown with different letters.

**Fig 5:**
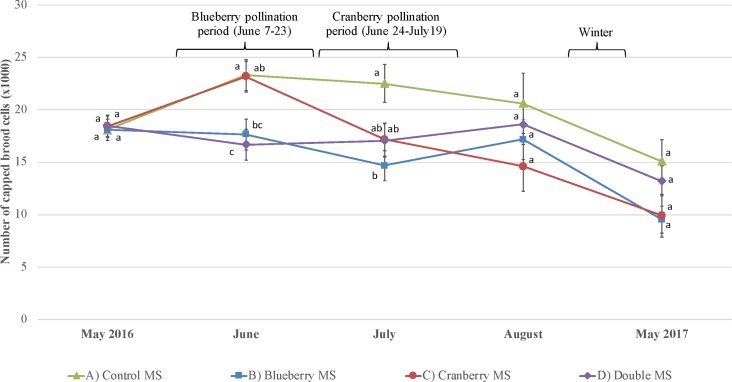
Surface of capped brood. Evolution of the surface of a honey bee colony capped brood (mean number of sealed brood cells ± SE) for each management strategy (n = 5 colonies per treatment) over a one-year period (from May 2016 to May 2017), including surveys before pollination, during blueberry pollination and cranberry pollination, after pollination and after wintering. Significant differences between treatments (p < 0.01 based on post hoc Tukey’s test—HSD) are shown with different letters.

Brood population: Results showed a significant difference between the different MS during the blueberry pollination (Fig 5): the surface of capped brood was lower for the Blueberry pollination and the double MS compared to the other groups (F_3,48_ = 5.72, p = 0.002). The surface of capped brood of colonies in the blueberry MS remained significantly lower than the control MS even after the blueberry pollination in July (F_3,48_ = 3.80, p = 0.02). All MS had similar brood population after the end of both pollination services (August and afterwards).

#### Pathogens and parasites

Virology: Larvae viruses’ prevalence (S2 Table): All colonies were absent of ABPV and CBPV during the whole study. Colonies were sporadically infected with DWV (less than 30%), IAPV (less than 40%) and KBV (less than 20%). Colonies were almost all infected with SBV (over 60%), except in May 2017 where no colony was infected. BQCV was present in all colonies, except one control MS colony in May 2017.

**Fig 6:**
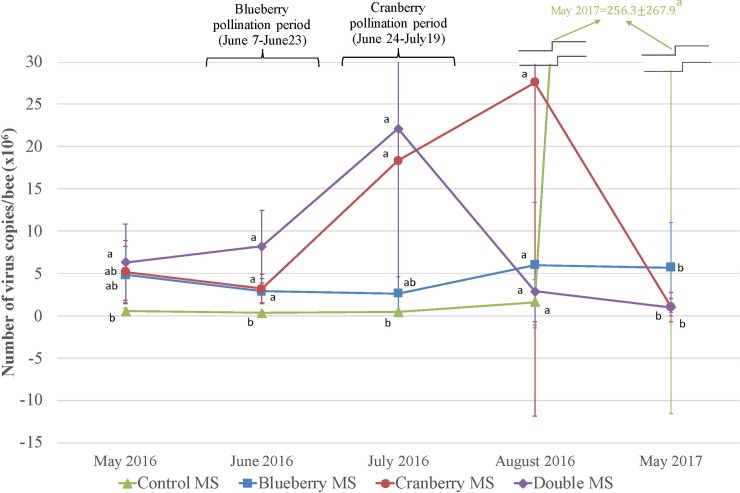
Black queen cell virus (BQCV) virulence. BQCV copies (mean number of copies±SE) diagnosed in honey bee larvae within the four management strategies (MS) (n = 5 colonies per MS), including surveys before pollination, during blueberry pollination and cranberry pollination, after pollination and after wintering. Significant differences among MS (p < 0.01 based on post hoc Tukey’s test—HSD) are shown with different letters.

**Fig 7:**
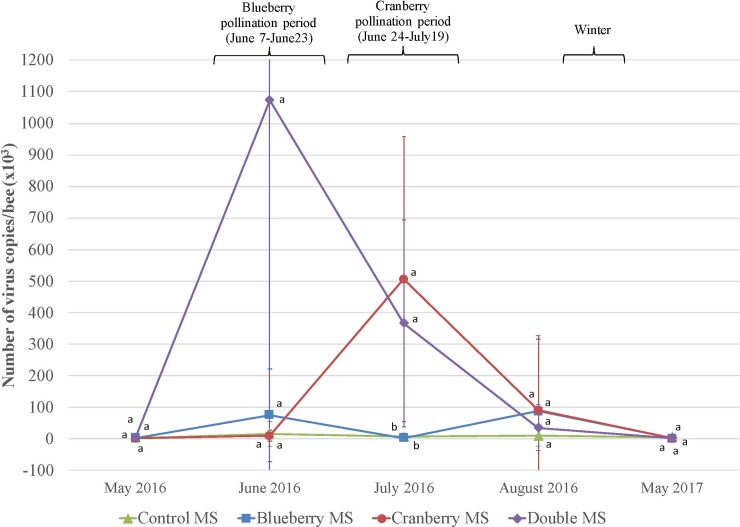
Sacbrood virus (SBV) virulence. SBV copies (mean number of copies ± SE) diagnosed in honey bee larvae within the four management strategies (MS) (n = 5 colonies per MS), including surveys before pollination, during blueberry pollination and cranberry pollination, after pollination and after wintering. Significant differences among MS (p < 0.01 based on post hoc Tukey’s test—HSD) are shown with different letters.

Larvae viruses’ virulence (S3 Table): Only BQCV and SBV showed significant differences in their number of copies per bee (F_12,52_ = 5.56, p<0.001 and F_10,34_ = 4.74, p<0.001) (Figs 6 and 7). Regarding BQCV, control MS had a significantly lower load of virus from May 2016 to August 2016 when all MS then showed statistically similar loads of BQCV (Fig 6). Despite, there is a tendency showing cranberry pollination services (cranberry MS and double MS in July 2016) was favorable for presence and multiplication of BQCV (Fig 6). We can’t explain the important difference seen in May 2017 for the control MS (F_3,52_ = 8.82, p<0.001) (Fig 6) as all the colonies from all MS have been wintered indoor together, moved in the same spring apiary in April 2017 and then moved in the farmland apiary in May 2017. There were significantly more SBV copies in larvae during the cranberry pollination (cranberry MS and double MS) (Fig 7).

Nurse bee viruses’ prevalence (S2 Table): All colonies were absent of ABPV during the whole study. Colonies were sporadically infected with CBPV (less than 40%), DWV (less than 30%) and KBV (up to 80% but most of them under 40%). Colonies were almost all infected with IAPV (most of them over 60%). BQCV and SBV were present in all colonies, except one double MS colony in May 2016 that showed presence of SBV.

Nurse bee viruses’ virulence (S4 Table): There was no significant difference among MS in the nurse bee virus’ number of copies.

Forager bee viruses’ prevalence (S2 Table): ABPV was rare (only one colony—within control MS—was infected with ABPV, and this in July 2016). CBPV and DWV were also rare (their presence was mostly below 20%). Colonies were sporadically infected with IAPV and KBV. BQCV was present in all colonies during the study, except in one colony in August 2016 within double MS. Finally, SBV infected generally over 80% of the colonies (S2 Table).

Forager bee viruses’ virulence (S5 Table): There was no significant difference among MS in the forager bee virus’ number of copies.

**Fig 8:**
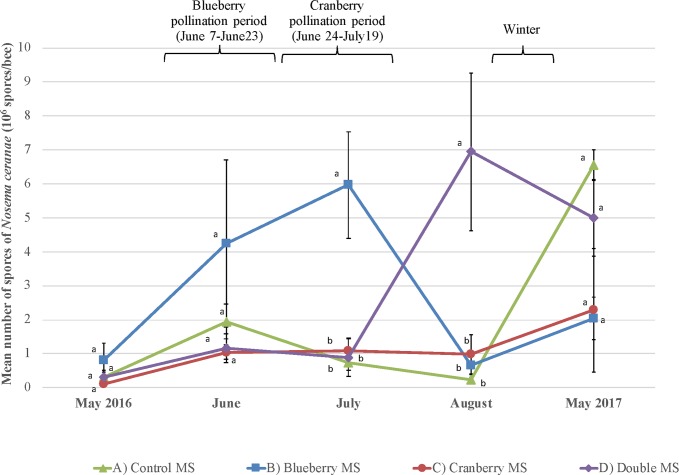
*Nosema ceranae* spores. Number of *Nosema ceranae* spores in honey bee gut (mean number of spores per bee ± SE) for each treatment (n = 5 colonies per treatment) over a one-year period (from May 2016 to May 2017), including surveys before pollination, during blueberry pollination and cranberry pollination, after pollination and after wintering. Significant differences between treatments (p < 0.01 based on post hoc Tukey’s test—HSD) are shown with different letters.

Nosemosis: *Nosema ceranae* infection levels are shown in Fig 8. They were significantly higher for the blueberry MS after the provision of blueberry pollination services on July 6 (F_3,53_ = 6.67, p<0.001) and for the double MS colonies in the farmland apiary after the provision of cranberry pollination services on August 17 (F_3,53_ = 10.49, p<0.001). *Nosema ceranae* infection levels returned to no significant differences the following spring.

*Varroa* mite: The *Varroa* mite infestation was low, i.e. under the economic thresholds of 1 fall/day in May and early June as well as 10 falls/day in July, August and September for all MS (S6 Table).

#### Queen loss

Colony swarming and queen loss were observed in nine colonies at some point during the study. Of these nine colonies, most were in the control MS and cranberry MS, mostly in June.

#### Winter mortality

The most important winter mortality occurred within the cranberry MS with 3 lost colonies (out of 5). Double and control MS lost 1 colony each, and blueberry MS did not lose any colony.

## Discussion

Crop pollination services with honey bees are important for lowbush blueberry and cranberry producers and represent a substantial source of income for beekeepers.

In this study, we followed honey bee colonies in four different pollination management strategies (MS): honey producing vs blueberry pollination and / or cranberry pollination. Our results show that pollination services in blueberry and cranberry crop landscapes have a negative impact on colonies, confirming the recent findings of other authors on other crops (apple, almond, cucumber, etc.): there is a reduced brood rearing [39], higher levels of pathogens [17, 56] and reduced lifespan [16]. Furthermore, [57] demonstrated that honey bees will collect a smaller amount nectar when floral ressources are scattered.

### Landscape foraging potential for honey bees

Our research shows that the blueberry apiary environment has good foraging potential for honey bees, similar to the farmland apiary. As expected, we found that the cranberry field of this study has insufficient resources to sustain the foraging needs of honey bees during cranberry pollination.

### Precipitation

Cranberry flowers produce considerably less nectar during rainy or windy days (Pedersen, 1953a in Cane and Schiffhauer [29]. In our study, there were 13 days of rain out of a total of 26 days during cranberry pollination services (as opposed to 5 days of rain at the control farmland site). During 6 rainy days, the cranberry site received more than 5 mm of rain. This phenomenon can partially explain the reduced nectar forage at this site. Furthermore, the cranberry fields were surrounded by poor forage areas. All these factors together may explain the low quantities of nectar collected by honey bees.

### Colony performance

#### Colony weight gain

The colonies in cranberry MS and double MS both suffered from a decline in weight gain due to a lack of nectar availability during cranberry pollination at our study site. Not long after the colonies arrived in the cranberry field, indeed, after one week, there was a significant difference in weight gain compared to the other groups. After two weeks, the difference was even greater. Even after returning to the farmland apiary, the colonies of these two MS never recovered from the cranberry nectar shortage. The lower colony weight gain had a carry-over effect after the provision of cranberry pollination services. At the end of the season, there was a difference of -25 kg to -45 kg in weight gain between the cranberry MS and the double MS compared to other groups. This phenomenon was also observed by Martin [58], who showed that honey bees pollinating cranberries needed a sucrose syrup supplement in order to offset the effects of a lack of nectar in that environment. This is also common knowledge among beekeepers who rent their colonies for cranberry pollination (Dufour, pers. obs.).

#### Brood population

The colonies of the two MS in the lowbush blueberry field had a significantly smaller capped brood area compared to the other groups. This is most likely an effect of a decline in egg-laying by the queen after a nutritional deficiency. Results from a previous study demonstrated that honey bees regulate brood population in response to the availability of pollen [59], and more recently, Girard *et al*. [31] found similar results. DeGrandi-Hoffman *et al*. [60] stressed that it is difficult to meet the nutritional requirements of managed colonies that offer pollination services, which leads to brood rearing scarcity. In another study with the same colonies and the same protocol (unpublished work), we found that pollen harvested by colonies during provision of pollination services to blueberries had significantly lower protein levels compared to pollen harvested by colonies located at the farmland apiary. Moreover, the protein level during blueberry pollination was lower than the minimal 20% of the pollen dryweight required for colony development and maintenance [61]. Those results may explain the significantly smaller capped brood area found in the colonies of the two MS who provided blueberry pollination services. However, brood population became similar for all MS after the end of pollination services in August which indicates a rebound of the queen laying once adverse conditions have settled.

#### Queen loss and colony winter mortality

The colonies with higher queen losses were located in the farmland apiary at the beginning of the season, among the control MS and cranberry MS. Neither of these colonies pollinated blueberry in June: they remained in what could be qualified as a better-quality environment. This caused the population to grow fast and strong, which led to a swarming. Thus, although these colonies should have been the strongest in this study for that time period, they were split, and their development delayed due to swarming. This incident certainly had an effect on the overall results of this study later on. Indeed, it may explain the lower weight gain of colonies in cranberry MS compared to the double MS colonies during and after the provision of cranberry pollination services, despite the fact that these colonies did not provide blueberry pollination services (Fig 4). Also, higher winter mortality occurred within the cranberry MS, most likely due to the aftermath of, first, swarming in June that led to smaller colonies, followed by lower nectar availability in July.

### Pathogens, parasites and viruses

Among the seven viruses analyzed in this study, ABPV, CBPV and DWV were those with less prevalence and virulence for the three castes (larvae, forager and nurse bees). Also, IAPV and KBV were rare in the larvae but generally present with important loads for the two other castes (forager and nurse bees). All these viruses did not show significant differences among MS throughout the study. The BQCV and SBV were the only viruses showing significant differences, especially for the control MS and both cranberry MS and double MS during the cranberry pollination services. The colonies in the cranberry MS and the double MS had indeed significantly higher levels of BQCV and SBV in July 2016 during cranberry pollination services. On the overall, results tend to show that the cranberry pollination services had an impact on the virulence of two viruses (BQCV and SBV) on honey bee colonies (Figs 6 and 7). This concurs with conclusions from other research, demonstrating the existence of a strong relationship between honey bee nutrition and their immunity against parasites and pathogens [62, 63]. Alaux *et al*. [21] and Erler *et al*. [64] demonstrated that monofloral diets reduce honey bee immunity in comparison with pollen that originates from polyfloral sources. This outcome can be translated to our experiment by comparing diets from pollination sites, especially cranberry fields, versus the farmland apiary, the latter, more diverse, probably enhancing honey bee immune functions thus reducing the prevalence of these viruses. However, mostly all colonies that survived returned to similar virus loads after the wintering which suggests no long-term effect of commercial pollination on honey bee colony virus infection.

Viral infections in bee colonies are often associated with *Varroa*, which acts as a vector [65], especially for the Deformed wing virus (DWV) [66, 67]. The consequences of virus-induced diseases are exacerbated by the presence of the *Varroa* mite and poor nutrition when bees are parasitized by the *Varroa* [68]. Colonies in our study had low levels of *Varroa* infestation, and this could explain the low virus loads measured in different MS throughout our experiment. Few experiments have studied the relationship between commercial pollination services provided by honey bees and virus prevalence and abundance. Glenny *et al*. [69] concluded honey bee health of commercially managed, migratory honey bee colonies is influenced by multiple biotic and abiotic factors and effects of pathogens on honey bee health must be investigated from a multidimensional approach. Smart et al. [18] compared colonies from a viral standpoint among other variables in different landscapes: those with higher overwintering survival were those surrounded by a better-quality forage during the summer, but they did not associate winter survival and viruses. Welch *et al*. [70] concluded it is important for the future of local and migratory honeybee colony health to continue to monitor and control these viruses and the diseases they cause as well as to identify newly emerging viruses.

*Nosema ceranae* infected all the colonies. Overall, the level of infection was low (below or just above the infection level of 1x10^6^ spores/bee) for most colonies at any point in time during the experiment. *Nosema* infection was slightly higher in the colonies in the blueberry MS in July, after blueberry pollination, and for the double MS in August, after cranberry pollination. These moderately infected colonies seem to suggest a relation with pollination services, but this has not been established conclusively in this study. *De facto*, nosemosis alone might not be the cause of honey bee mortality or diminution in productivity and population development, but it could certainly have participated in a synergistic relationship with other harmful factors to engender higher colony mortality [71]. Moreover, following the example of viruses, colonies from all MS regained similar *Nosema ceranae* infection level the following spring.

In summary, our goal was to answer one main question: Do floral abundance and attractiveness during provision of lowbush blueberry and cranberry pollination services have an impact on honey bee colony health and development? Our study shows that the apiary situated in the cranberry monoculture, with its low proportion of attractive forage in the vicinity, experienced a negative impact on colony health status. Colony weight gain did not increase during the cranberry pollination period compared to colonies managed in the farmland apiary. This result is alarming, especially since this period corresponds to the best honey flow period. Even after colonies had been returned to the farmland apiary, they failed to store honey and suffered a carry-over effect. The impact of lowbush blueberry pollination on colonies is less obvious. In spite of the diversity and attractiveness of flowers surrounding the blueberry apiary and colony weight gain, the brood population was reduced. This result is corroborated by an unpublished 2-year study (from our research team) in the same conditions: protein concentration in the blueberry apiary diversified pollen collected by the honey bees was lower than the recommended threshold of 20% found by Kleinschmidt *et al*. [61] for normal development of colonies, which can explain the significant lower brood area of blueberry MS and double MS during the blueberry pollination. The blueberry MS was able to recover after deployment of pollination services, but colonies in the double MS seemed more affected and showed significantly more viral infections than in the colonies that only pollinated cranberries. It would be interesting in the future to test different sites for the same crop. This would help explain the cause of nutritional differences and their link to either the pollination services or the crop environment. Because crop pollination services using honey bees are important for lowbush blueberry and cranberry producers and represent a substantial source of income for beekeepers, and because all questions have not been answered yet, it is important to try to mitigate the impact of the commercial pollination on honey bee colonies. To that effect, we suggest feeding the colonies with high protein concentration pollen patties during the provision of blueberry pollination services and sucrose syrup during the provision of cranberry pollination services in order to mitigate the impacts of those activities on the honey bee colonies. More research is needed to assess the impacts of commercial pollination of lowbush blueberry and cranberry on honey bee health, especially on the synergetic effects of forage availability and quality, pathogens and honey bee management.

## Supporting information

S1 Table List of the different land types within the three honey bee foraging radii (1.5K, 3K and 5K) for each apiary (farmland, blueberry and cranberry apiaries). (XLSX)

S2 Table Virus prevalence (presence or absence) per honey bee colony for the 7 viruses analyzed within three castes (larvae, nurse and forager bees) of each management strategy throughout the study. (PDF)

S3 Table Virus virulence (mean virus copies per bee) per honey bee colony for the 7 viruses analyzed within larvae of each management strategy throughout the study. (PDF)

S4 Table Virus virulence (mean virus copies per bee) per honey bee colony for the 7 viruses analyzed within nurse bees of each management strategy throughout the study. (PDF)

S5 Table Virus virulence (mean virus copies per bee) per honey bee colony for the 7 viruses analyzed within forager bees of each management strategy throughout the study. (PDF)

S6 Table *Varroa* mite population monitoring. (PDF)

S1 Fig Hive weight gain (mean weight in kilograms ±SE) per treatment (N = 5 colonies per treatment) over a season (from June 1, 2016 to September 6, 2016), including surveys before pollination, during blueberry pollination and cranberry pollination and after pollination. Significant differences among treatments (P< 0.01 based on post hoc Tukey’s test -HSD) are shown with different letters.
(XLSX)

S2 Fig Evolution of the surface of a honey bee colony capped brood (mean number of sealed brood cells ±SE) for each management strategy (N = 5 colonies per treatment) over a one-year period (from May 2016 to May 2017), including surveys before pollination, during blueberry pollination and cranberry pollination, after pollination and after wintering. Significant differences among treatments (P< 0.01 based on post hoc Tukey’s test -HSD) are shown with different letters
(XLSX)

S3 Fig Virology analysis Black queen cell virus (BQCV) and Sacbrood virus (SBV) virulence. Virus copies (mean number of copies ±SE) diagnosed in honey bee larvae within the four management strategies (MS) (N = 5 colonies per MS), including surveys before pollination, during blueberry pollination and cranberry pollination, after pollination and after wintering. Significant differences among MS (P< 0.01 based on post hoc Tukey’s test -HSD) are shown with different letters.
(XLSX)

S4 Fig *Nosema ceranae* spores. Number of *Nosema ceranae* spores in honey bee gut (mean number of spores per bee ±SE) for each treatment (N = 5 colonies per treatment) over a one-year period (from May 2016 to May 2017), including surveys before pollination, during blueberry pollination and cranberry pollination, after pollination and after wintering. Significant differences among treatments (P< 0.01 based on post hoc Tukey’s test -HSD) are shown with different letters.
(XLSX)
