# Supplementary material for: The impact of lowbush blueberry (Vaccinium angustifolium Ait.) and cranberry (Vaccinium macrocarpon Ait.) pollination on honey bee (Apis mellifera L.) colony health status
Source: PLoS One. 2020 Jan 24;15(1):e0227970. doi: 10.1371/journal.pone.0227970 (PMC6980599; doi:10.1371/journal.pone.0227970)
Supplement: S2 Table — (PDF) [file pone.0227970.s002.pdf]

		Acute bee paralysis virus (ABPV)					Black queen cell virus (BQCV)					Chronic bee paralysis virus (CBPV)					Deformed wing virus (DWV)					Israeli acute paralysis virus (IAPV)					Kashmir bee virus (KBV)					Sac brood virus (SBV)				
Colony #	Management Strategies (MS)	Larvae					Larvae					Larvae					Larvae					Larvae					Larvae					Larvae				
		May 2016	June	July	August	May 2017	May 2016	June	July	August	May 2017	May 2016	June	July	August	May 2017	May 2016	June	July	August	May 2017	May 2016	June	July	August	May 2017	May 2016	June	July	August	May 2017	May 2016	June	July	August	May 2017
206		-	-	N/A*	-	-	+	+	N/A*	+	+	-	-	N/A*	-	-	-	-	N/A*	-	-	-	-	N/A*	-	-	-	-	N/A*	-	-	+	+	N/A*	+	+
221		-	-	-	N/A*	-	+	+	+	N/A*	+	-	-	-	N/A*	-	-	-	-	N/A*	-	-	-	-	N/A*	-	-	-	-	N/A	-	+	+	+	N/A*	-
378	Control MS	-	-	-	-	Dead[1]	+	+	+	+	Dead[1]	-	-	-	-	Dead[1]	-	-	-	-	Dead[1]	-	+	+	-	Dead[1]	-	+	-	-	Dead[1]	-	-	-	+	Dead[1]
500		-	-	-	-	-	+	+	+	+	-	-	-	-	-	-	-	-	-	-	+	-	-	-	-	-	-	-	-	-	-	+	+	+	-	-
574		-	-	N/A*	-	-	+	+	N/A*	+	+	-	-	N/A*	-	-	-	-	N/A*	-	-	-	-	N/A*	-	-	-	-	N/A*	-	-	-	+	N/A*	+	+
316		-	-	-	-	-	+	+	+	+	+	-	-	-	-	-	-	-	+	-	-	-	-	-	-	-	-	-	-	-	-	+	+	+	+	-
361		-	-	-	-	-	+	+	+	+	+	-	-	-	-	-	-	-	-	-	-	+	-	-	-	-	+	-	-	-	-	-	+	+	+	-
469	Blueberry MS	-	-	-	-	-	+	+	+	+	+	-	-	-	-	-	-	-	-	-	-	+	+	+	-	-	-	-	-	-	-	+	+	+	-	-
582		-	-	-	-	-	+	+	+	+	+	-	-	-	-	-	-	-	-	-	-	-	-	-	-	-	-	-	-	-	-	+	+	+	+	-
596		-	-	-	-	-	+	+	+	+	+	-	-	-	-	-	-	-	-	-	-	-	+	-	-	-	-	-	-	-	-	-	+	+	+	-
485		-	-	-	-	Dead[1]	+	+	+	+	Dead[1]	-	-	-	-	Dead[1]	-	-	+	-	Dead[1]	-	-	-	-	Dead[1]	-	-	-	-	Dead[1]	+	+	+	+	Dead[1]
492		-	-	-	N/A*	Dead[1]	+	+	+	N/A*	Dead[1]	-	-	-	N/A*	Dead[1]	-	-	-	N/A*	Dead[1]	+	-	-	N/A*	Dead[1]	+	-	-	N/A*	Dead[1]	+	+	+	N/A*	Dead[1]
573	Cranberry MS	-	-	N/A*	-	-	+	+	N/A*	+	+	-	-	N/A*	-	-	-	-	N/A*	-	-	-	-	N/A*	+	-	-	-	N/A*	-	-	+	+	N/A*	+	-
578		-	-	-	-	-	+	+	+	+	+	-	-	-	-	-	-	-	+	-	-	-	-	-	-	-	-	-	-	-	-	+	+	+	+	-
587		-	-	-	-	Dead[1]	+	+	+	+	Dead[1]	-	-	-	-	Dead[1]	-	-	+	-	Dead[1]	-	-	-	-	Dead[1]	-	-	-	-	Dead[1]	+	+	+	+	Dead[1]
200		-	-	N/A*	-	Dead[1]	+	+	N/A*	+	Dead[1]	-	-	N/A*	-	Dead[1]	-	-	N/A*	-	Dead[1]	-	+	N/A*	-	Dead[1]	-	-	N/A*	-	Dead[1]	+	+	N/A*	+	Dead[1]
488		-	-	-	-	-	+	+	+	+	+	-	-	-	-	-	-	-	-	-	-	-	-	-	+	-	-	-	-	-	-	-	+	+	+	-
516	Double MS	-	-	-	-	-	+	+	+	+	+	-	-	-	-	-	-	-	+	-	-	-	+	-	+	-	-	-	-	-	-	+	+	+	+	-
546		-	-	-	-	-	+	+	+	+	+	-	-	-	-	-	-	-	-	-	-	+	-	-	-	-	-	-	-	-	-	+	+	+	+	-
598		-	-	-	-	-	+	+	+	+	+	-	-	-	-	-	-	-	-	-	-	-	-	-	-	-	-	-	-	-	-	+	+	+	+	+
		Acute bee paralysis virus (ABPV)					Black queen cell virus (BQCV)					Chronic bee paralysis virus (CBPV)					Deformed wing virus (DWV)					Israeli acute paralysis virus (IAPV)					Kashmir bee virus (KBV)					Sac brood virus (SBV)				
Colony #	Management Strategies (MS)	Nurse					Nurse					Nurse					Nurse					Nurse					Nurse					Nurse				
		May 2016	June	July	August	May 2017	May 2016	June	July	August	May 2017	May 2016	June	July	August	May 2017	May 2016	June	July	August	May 2017	May 2016	June	July	August	May 2017	May 2016	June	July	August	May 2017	May 2016	June	July	August	May 2017
206		-	-	-	-	-	+	+	+	+	+	-	-	-	+	+	-	+	-	-	+	+	+	+	+	+	+	+	+	-	-	+	+	+	+	+
221		-	-	-	-	-	+	+	+	+	+	+	+	+	+	+	+	+	+	-	+	-	+	-	-	-	+	+	-	-	-	+	+	+	+	+
378	Control MS	-	-	-	-	Dead[1]	+	+	+	+	Dead[1]	-	-	-	-	Dead[1]	-	+	-	-	Dead[1]	+	+	+	-	Dead[1]	+	+	+	-	Dead[1]	+	+	+	+	Dead[1]
500		-	-	-	+	-	+	+	+	+	+	-	-	-	+	-	-	-	-	-	-	-	+	-	+	-	-	+	-	-	-	+	+	+	+	+
574		-	-	-	-	-	+	+	+	+	+	-	-	-	-	-	-	-	+	-	+	-	+	+	-	-	-	-	-	-	-	+	+	+	+	+
316		-	-	-	-	-	+	+	+	+	+	-	-	-	-	-	-	-	-	-	-	+	+	+	+	+	+	-	-	-	-	+	+	+	+	+
361		-	-	-	-	-	+	+	+	+	+	-	-	-	-	+	-	-	-	+	+	+	-	-	-	-	+	-	-	-	-	+	+	+	+	+
469	Blueberry MS	-	-	-	-	-	+	+	+	+	+	-	-	-	-	-	-	-	-	+	+	+	+	-	-	+	+	-	-	-	-	+	+	+	+	+
582		-	-	-	-	-	+	+	+	+	+	-	-	-	+	-	-	-	-	-	-	+	+	+	-	-	+	+	-	-	-	+	+	+	+	+
596		-	-	-	-	-	+	+	+	+	+	-	-	-	-	-	-	-	-	-	-	+	+	+	+	-	+	+	-	-	-	+	+	+	+	+
485		-	-	-	-	Dead[1]	+	+	+	+	Dead[1]	-	+	-	-	Dead[1]	-	-	+	+	Dead[1]	-	+	-	+	Dead[1]	-	+	-	-	Dead[1]	+	+	+	+	Dead[1]
492		-	-	-	-	Dead[1]	+	+	+	+	Dead[1]	-	-	-	-	Dead[1]	-	-	-	-	Dead[1]	+	+	+	+	Dead[1]	-	+	-	+	Dead[1]	+	+	+	+	Dead[1]
573	Cranberry MS	-	-	-	-	+	+	+	+	+	+	-	-	-	-	-	-	-	+	-	+	-	+	+	+	-	+	+	-	+	-	+	+	+	+	+
578		-	-	-	-	-	+	+	+	+	+	-	-	-	-	-	-	-	-	-	-	-	+	+	+	-	-	+	-	+	-	+	+	+	+	+
587		-	-	-	-	Dead[1]	+	+	+	+	Dead[1]	-	-	-	-	Dead[1]	-	-	-	-	Dead[1]	-	-	+	-	Dead[1]	-	-	-	-	Dead[1]	+	+	+	+	Dead[1]
200		-	-	-	-	Dead[1]	+	+	+	+	Dead[1]	-	-	-	+	Dead[1]	-	-	+	-	Dead[1]	-	+	+	-	Dead[1]	-	+	-	-	Dead[1]	+	+	+	+	Dead[1]
488		-	-	-	-	-	+	+	+	+	+	-	-	-	-	-	-	-	+	-	-	+	+	+	+	-	+	+	+	+	-	+	+	+	+	+
516	Double MS	-	-	-	-	-	+	+	+	+	+	-	-	-	-	-	-	+	+	-	+	+	+	+	+	-	+	+	+	+	-	+	+	+	+	+
546		-	-	-	-	-	+	+	+	+	+	-	-	-	-	+	-	-	-	+	+	+	+	+	+	-	+	-	+	-	-	+	+	+	+	+
598		-	-	-	-	-	+	+	+	+	+	+	-	-	-	-	-	-	-	-	-	+	+	+	+	+	-	+	+	-	+	+	+	+	+	+
		Acute bee paralysis virus (ABPV)					Black queen cell virus (BQCV)					Chronic bee paralysis virus (CBPV)					Deformed wing virus (DWV)					Israeli acute paralysis virus (IAPV)					Kashmir bee virus (KBV)					Sac brood virus (SBV)				
Colony #	Management Strategies (MS)	Forager					Forager					Forager					Forager					Forager					Forager					Forager				
		May 2016	June	July	August	May 2017	May 2016	June	July	August	May 2017	May 2016	June	July	August	May 2017	May 2016	June	July	August	May 2017	May 2016	June	July	August	May 2017	May 2016	June	July	August	May 2017	May 2016	June	July	August	May 2017
206		-	-	-	-	-	+	+	+	+	+	+	-	+	-	+	+	-	-	-	-	+	+	+	+	-	-	+	+	+	-	+	+	+	+	+
221		-	-	+	-	-	+	+	+	+	+	-	-	+	+	-	-	-	-	-	-	+	+	+	-	-	+	-	+	-	-	+	+	+	+	+
378	Control MS	-	-	-	-	Dead[1]	+	+	+	+	Dead[1]	-	-	+	+	Dead[1]	-	+	-	-	Dead[1]	+	+	+	+	Dead[1]	-	+	+	-	Dead[1]	+	+	+	+	Dead[1]
500		-	-	-	-	-	+	+	+	+	+	+	-	+	+	-	-	-	-	-	-	+	+	+	-	-	-	-	-	-	-	+	+	+	+	+
574		-	-	-	-	-	+	+	+	+	+	-	-	-	-	-	-	-	+	-	-	-	+	+	-	-	-	+	+	-	-	-	+	+	+	+
316		-	-	-	-	-	+	+	+	+	+	+	-	+	-	+	-	-	-	-	-	+	+	+	-	+	-	+	-	-	-	+	+	+	+	+
361		-	-	-	-	-	+	+	+	+	+	+	-	+	-	-	-	-	-	+	-	+	+	-	+	-	+	+	-	-	-	+	+	+	+	+
469	Blueberry MS	-	-	-	-	-	+	+	+	+	+	-	+	+	+	-	-	-	-	-	-	+	+	-	+	-	+	-	-	-	-	+	+	+	+	+
582		-	-	+	-	-	+	+	+	+	+	-	-	+	-	+	-	-	-	-	-	-	-	-	-	+	-	-	-	-	-	+	+	+	+	-
596		-	-	-	-	-	+	+	+	+	+	+	-	-	-	-	-	-	-	-	-	+	+	+	-	-	+	-	-	-	-	+	+	+	+	+
485		-	-	-	-	Dead[1]	+	+	+	+	Dead[1]	-	-	-	+	Dead[1]	-	-	-	-	Dead[1]	-	+	+	-	Dead[1]	-	+	-	-	Dead[1]	+	+	+	+	Dead[1]
492		-	-	-	-	Dead[1]	+	+	+	+	Dead[1]	-	-	+	-	Dead[1]	-	-	-	-	Dead[1]	-	+	+	+	Dead[1]	+	+	-	+	Dead[1]	+	+	+	+	Dead[1]
573	Cranberry MS	-	-	-	-	-	+	+	+	+	+	-	-	-	+	-	-	-	+	-	-	-	+	-	+	-	-	+	-	-	-	+	+	+	+	+
578		-	-	-	-	-	+	+	+	+	+	+	-	+	+	+	-	-	-	-	-	-	-	+	+	-	-	-	+	+	-	+	+	+	+	-
587		-	-	-	-	Dead[1]	+	+	+	+	Dead[1]	-	+	-	+	Dead[1]	-	+	-	-	Dead[1]	-	+	+	+	Dead[1]	-	+	+	-	Dead[1]	+	+	+	+	Dead[1]
200		-	-	-	-	Dead[1]	+	+	+	+	Dead[1]	-	-	+	+	Dead[1]	+	-	-	-	Dead[1]	-	+	+	+	Dead[1]	-	+	-	-	Dead[1]	+	+	+	+	Dead[1]
488		-	-	-	-	-	+	+	+	+	+	+	-	-	+	-	+	-	-	-	-	+	+	+	+	-	+	+	+	-	-	+	+	+	+	-
516	Double MS	-	-	-	-	-	+	+	+	+	+	-	-	+	-	-	-	+	-	-	-	+	+	+	+	-	-	+	+	+	-	+	+	+	+	+
546		-	-	-	-	-	+	+	+	+	+	+	-	-	+	-	-	+	-	-	+	+	+	+	+	-	+	+	+	-	-	+	+	+	+	-
598		-	-	-	-	-	+	+	+	+	+	+	-	+	+	+	-	-	+	+	-	-	+	+	+	-	-	-	+	-	-	+	+	+	+	+

"+" indicates the virus was detected and "-" indicates the virus was not present

* N/A indicates there was no larvae in the colony at the moment of the sampling

[1] "Dead" indicates the colony has died over the winter 2016-2017
