# Supplementary material for: The impact of lowbush blueberry (Vaccinium angustifolium Ait.) and cranberry (Vaccinium macrocarpon Ait.) pollination on honey bee (Apis mellifera L.) colony health status
Source: PLoS One. 2020 Jan 24;15(1):e0227970. doi: 10.1371/journal.pone.0227970 (PMC6980599; doi:10.1371/journal.pone.0227970)
Supplement: S3 Table — (PDF) [file pone.0227970.s003.pdf]

BEEHIVE#	MANAGEMENT STRATEGIES	TIME	APIARY	VIRUSES IN LARVAE						
				ABPV	BQCV	CBPV	DWV	IAPV	KBV	SBV
206	CONTROL MS	MAY 2016	FARMLAND	0	218867	0	0	0	0	361
221	CONTROL MS	MAY 2016	FARMLAND	0	761066	0	0	0	0	1524
378	CONTROL MS	MAY 2016	FARMLAND	0	716367	0	0	0	0	0
500	CONTROL MS	MAY 2016	FARMLAND	0	917468	0	0	0	0	2830
574	CONTROL MS	MAY 2016	FARMLAND	0	104111	0	0	0	0	0
316	BLUEBERRY MS	MAY 2016	FARMLAND	0	4038665	0	0	0	0	1865
361	BLUEBERRY MS	MAY 2016	FARMLAND	0	22740457	0	0	36223	23409	0
469	BLUEBERRY MS	MAY 2016	FARMLAND	0	9658928	0	0	2600	0	949
582	BLUEBERRY MS	MAY 2016	FARMLAND	0	447917	0	0	0	0	381
596	BLUEBERRY MS	MAY 2016	FARMLAND	0	2268965	0	0	0	0	0
485	CRANBERRY MS	MAY 2016	FARMLAND	0	6605655	0	0	0	0	207
492	CRANBERRY MS	MAY 2016	FARMLAND	0	33564073	0	0	<MDL[2]	<MDL[2]	375
573	CRANBERRY MS	MAY 2016	FARMLAND	0	1570629	0	0	0	0	263
578	CRANBERRY MS	MAY 2016	FARMLAND	0	2142342	0	0	0	0	3204
587	CRANBERRY MS	MAY 2016	FARMLAND	0	1732777	0	0	0	0	3196
200	DOUBLE MS	MAY 2016	FARMLAND	0	379520	0	0	0	0	230
488	DOUBLE MS	MAY 2016	FARMLAND	0	8761661	0	0	0	0	0
516	DOUBLE MS	MAY 2016	FARMLAND	0	3574647	0	0	0	0	5364
546	DOUBLE MS	MAY 2016	FARMLAND	0	3869747	0	0	4876	0	134
598	DOUBLE MS	MAY 2016	FARMLAND	0	76246850	0	0	0	0	5515
206	CONTROL MS	JUNE 2016	FARMLAND	0	326789	0	0	0	0	13325
221	CONTROL MS	JUNE 2016	FARMLAND	0	167318	0	0	0	0	58318
378	CONTROL MS	JUNE 2016	FARMLAND	0	124129	0	0	150513	2127	0
500	CONTROL MS	JUNE 2016	FARMLAND	0	441203	0	0	0	0	893
574	CONTROL MS	JUNE 2016	FARMLAND	0	808149	0	0	0	0	1448
316	BLUEBERRY MS	JUNE 2016	BLUEBERRY	0	4879451	0	0	0	0	53221
361	BLUEBERRY MS	JUNE 2016	BLUEBERRY	0	4367077	0	0	0	0	1558302
469	BLUEBERRY MS	JUNE 2016	BLUEBERRY	0	5255496	0	0	4373	0	82519
582	BLUEBERRY MS	JUNE 2016	BLUEBERRY	0	1951088	0	0	0	0	5521
596	BLUEBERRY MS	JUNE 2016	BLUEBERRY	0	506147	0	0	4579	0	1074
485	CRANBERRY MS	JUNE 2016	FARMLAND	0	3278469	0	0	0	0	23317
492	CRANBERRY MS	JUNE 2016	FARMLAND	0	28433530	0	0	0	0	7390
573	CRANBERRY MS	JUNE 2016	FARMLAND	0	1788415	0	0	0	0	6627
578	CRANBERRY MS	JUNE 2016	FARMLAND	0	1062109	0	0	0	0	453
587	CRANBERRY MS	JUNE 2016	FARMLAND	0	1025490	0	0	0	0	2266
200	DOUBLE MS	JUNE 2016	BLUEBERRY	0	8415389	0	0	400191	0	190228733
488	DOUBLE MS	JUNE 2016	BLUEBERRY	0	2841796	0	0	0	0	62799
516	DOUBLE MS	JUNE 2016	BLUEBERRY	0	3934868	0	0	20950	0	21260
546	DOUBLE MS	JUNE 2016	BLUEBERRY	0	4432069	0	0	0	0	16580
598	DOUBLE MS	JUNE 2016	BLUEBERRY	0	48822729	0	0	0	0	6186383
206	CONTROL MS	JULY 2016	FARMLAND	N/A[1]	N/A[1]	N/A[1]	N/A[1]	N/A[1]	N/A[1]	N/A[1]
221	CONTROL MS	JULY 2016	FARMLAND	0	725668	0	0	0	0	8573
378	CONTROL MS	JULY 2016	FARMLAND	0	79847	0	0	4161983	0	0
500	CONTROL MS	JULY 2016	FARMLAND	0	432541	0	0	0	0	1507
574	CONTROL MS	JULY 2016	FARMLAND	N/A[1]	N/A[1]	N/A[1]	N/A[1]	N/A[1]	N/A[1]	N/A[1]
316	BLUEBERRY MS	JULY 2016	FARMLAND	0	5206194	0	<MDL[2]	0	0	1093
361	BLUEBERRY MS	JULY 2016	FARMLAND	0	1573081	0	0	0	0	2896
469	BLUEBERRY MS	JULY 2016	FARMLAND	0	2339400	0	0	5949	0	17473
582	BLUEBERRY MS	JULY 2016	FARMLAND	0	805981	0	0	0	0	195
596	BLUEBERRY MS	JULY 2016	FARMLAND	0	2448360	0	0	0	0	1972
485	CRANBERRY MS	JULY 2016	CRANBERRY	0	8356979	0	<MDL[2]	0	0	232042
492	CRANBERRY MS	JULY 2016	CRANBERRY	0	7422035	0	0	0	0	87901
573	CRANBERRY MS	JULY 2016	CRANBERRY	N/A[1]	N/A[1]	N/A[1]	N/A[1]	N/A[1]	N/A[1]	N/A[1]
578	CRANBERRY MS	JULY 2016	CRANBERRY	0	5896345	0	<MDL[2]	0	0	216004
587	CRANBERRY MS	JULY 2016	CRANBERRY	0	118367260	0	<MDL[2]	0	0	4186770
200	DOUBLE MS	JULY 2016	CRANBERRY	N/A[1]	N/A[1]	N/A[1]	N/A[1]	N/A[1]	N/A[1]	N/A[1]
488	DOUBLE MS	JULY 2016	CRANBERRY	0	3811996	0	0	0	0	281093
516	DOUBLE MS	JULY 2016	CRANBERRY	0	4410712	0	<MDL[2]	0	0	36916
546	DOUBLE MS	JULY 2016	CRANBERRY	0	4267112	0	0	0	0	157071
598	DOUBLE MS	JULY 2016	CRANBERRY	0	1035838823	0	0	0	0	1858899
206	CONTROL MS	AUGUST 2016	FARMLAND	0	237947	0	0	0	0	174
221	CONTROL MS	AUGUST 2016	FARMLAND	N/A[1]	N/A[1]	N/A[1]	N/A[1]	N/A[1]	N/A[1]	N/A[1]
378	CONTROL MS	AUGUST 2016	FARMLAND	0	1012487	0	0	0	0	13651
500	CONTROL MS	AUGUST 2016	FARMLAND	0	<MDL[2]	0	0	0	0	0
574	CONTROL MS	AUGUST 2016	FARMLAND	0	769970	0	0	0	0	4230
316	BLUEBERRY MS	AUGUST 2016	FARMLAND	0	2465875	0	0	0	0	9534
361	BLUEBERRY MS	AUGUST 2016	FARMLAND	0	12352314	0	0	0	0	71950
469	BLUEBERRY MS	AUGUST 2016	FARMLAND	0	861945	0	0	0	0	0
582	BLUEBERRY MS	AUGUST 2016	FARMLAND	0	13768075	0	0	0	0	23459
596	BLUEBERRY MS	AUGUST 2016	FARMLAND	0	2138599	0	0	0	0	27379
485	CRANBERRY MS	AUGUST 2016	FARMLAND	0	2463063	0	0	0	0	31114
492	CRANBERRY MS	AUGUST 2016	FARMLAND	N/A[1]	N/A[1]	N/A[1]	N/A[1]	N/A[1]	N/A[1]	N/A[1]
573	CRANBERRY MS	AUGUST 2016	FARMLAND	0	21875315	0	0	<MDL[2]	0	50602
578	CRANBERRY MS	AUGUST 2016	FARMLAND	0	1865922	0	0	0	0	11238
587	CRANBERRY MS	AUGUST 2016	FARMLAND	0	936344846	0	0	0	0	96001
200	DOUBLE MS	AUGUST 2016	FARMLAND	0	1826084	0	0	0	0	23454
488	DOUBLE MS	AUGUST 2016	FARMLAND	0	140734	0	0	8823	0	196
516	DOUBLE MS	AUGUST 2016	FARMLAND	0	1024117	0	0	6169	0	71402
546	DOUBLE MS	AUGUST 2016	FARMLAND	0	375814	0	0	0	0	185
598	DOUBLE MS	AUGUST 2016	FARMLAND	0	217684760	0	0	0	0	12995651
206	CONTROL MS	MAY 2017	FARMLAND	0	1175000000	0	0	0	0	5363
221	CONTROL MS	MAY 2017	FARMLAND	0	1240000000	0	0	0	0	0
378	CONTROL MS	MAY 2017	FARMLAND	Dead colony						
500	CONTROL MS	MAY 2017	FARMLAND	0	62000000	0	0	0	0	0
574	CONTROL MS	MAY 2017	FARMLAND	0	10000000	0	0	0	0	2583
316	BLUEBERRY MS	MAY 2017	FARMLAND	0	840000	0	0	0	0	0
361	BLUEBERRY MS	MAY 2017	FARMLAND	0	26650000	0	0	0	0	0
469	BLUEBERRY MS	MAY 2017	FARMLAND	0	1675000	0	0	0	0	0
582	BLUEBERRY MS	MAY 2017	FARMLAND	0	5150000	0	0	0	0	0
596	BLUEBERRY MS	MAY 2017	FARMLAND	0	6900000	0	0	0	0	0
485	CRANBERRY MS	MAY 2017	FARMLAND	Dead colony						
492	CRANBERRY MS	MAY 2017	FARMLAND	Dead colony						
573	CRANBERRY MS	MAY 2017	FARMLAND	0	1785000	0	0	0	0	0
578	CRANBERRY MS	MAY 2017	FARMLAND	0	75500	0	0	0	0	0
587	CRANBERRY MS	MAY 2017	FARMLAND	Dead colony						
200	DOUBLE MS	MAY 2017	FARMLAND	Dead colony						
488	DOUBLE MS	MAY 2017	FARMLAND	0	417000	0	0	0	0	0
516	DOUBLE MS	MAY 2017	FARMLAND	0	945000	0	0	0	0	0
546	DOUBLE MS	MAY 2017	FARMLAND	0	1110000	0	0	0	0	0
598	DOUBLE MS	MAY 2017	FARMLAND	0	500000	0	0	0	0	1162

[1] "N/A" indicates larvae were not available in a given colony at the sampling time

[2] "<MDL" indicates the number of virus copies was below the minimum detection limit but was positive
