# Supplementary material for: The impact of lowbush blueberry (Vaccinium angustifolium Ait.) and cranberry (Vaccinium macrocarpon Ait.) pollination on honey bee (Apis mellifera L.) colony health status
Source: PLoS One. 2020 Jan 24;15(1):e0227970. doi: 10.1371/journal.pone.0227970 (PMC6980599; doi:10.1371/journal.pone.0227970)
Supplement: S4 Table — (PDF) [file pone.0227970.s004.pdf]

BEEHIVE#	MANAGEMENT STRATEGIES	TIME	APIARY	VIRUSES IN NURSE BEES						
				ABPV	BQCV	CBPV	DWV	IAPV	KBV	SBV
206	CONTROL MS	MAY 2016	FARMLAND	0	887222126	0	0	2680	11293	172950
221	CONTROL MS	MAY 2016	FARMLAND	0	45274237870	<MDL[1]	<MDL[1]	0	33682	118443853100
378	CONTROL MS	MAY 2016	FARMLAND	0	222904710	0	0	1130000	1102706	55263
500	CONTROL MS	MAY 2016	FARMLAND	0	218706721	0	0	0	0	89121
574	CONTROL MS	MAY 2016	FARMLAND	0	75159554	0	0	0	0	1229
316	BLUEBERRY MS	MAY 2016	FARMLAND	0	533919462	0	0	2480000	191619	24093601
361	BLUEBERRY MS	MAY 2016	FARMLAND	0	366664372	0	0	45300	7280	507899729000
469	BLUEBERRY MS	MAY 2016	FARMLAND	0	477106712	0	0	39300000	439102	53008
582	BLUEBERRY MS	MAY 2016	FARMLAND	0	124295866	0	0	87500	<MDL[1]	378594
596	BLUEBERRY MS	MAY 2016	FARMLAND	0	168347381	0	0	1045000	8512	10635
485	CRANBERRY MS	MAY 2016	FARMLAND	0	599269083	0	0	0	0	3825
492	CRANBERRY MS	MAY 2016	FARMLAND	0	12237867615	0	0	<MDL[1]	0	21439
573	CRANBERRY MS	MAY 2016	FARMLAND	0	51729492	0	0	0	<MDL[1]	25536
578	CRANBERRY MS	MAY 2016	FARMLAND	0	36525741	0	0	0	0	1228
587	CRANBERRY MS	MAY 2016	FARMLAND	0	359943006	0	0	0	0	8444
200	DOUBLE MS	MAY 2016	FARMLAND	0	157087654	0	0	0	0	26614
488	DOUBLE MS	MAY 2016	FARMLAND	0	502457937	0	0	625000000	8809419	494679
516	DOUBLE MS	MAY 2016	FARMLAND	0	305148498	0	0	7050000	153604	125038417600
546	DOUBLE MS	MAY 2016	FARMLAND	0	120607137	0	0	1015000	<MDL[1]	<MDL[1]
598	DOUBLE MS	MAY 2016	FARMLAND	0	617232264000	<MDL[1]	0	<MDL[1]	0	268216
206	CONTROL MS	JUNE 2016	FARMLAND	0	416189549300	0	<MDL[1]	265000000	59561093	135862
221	CONTROL MS	JUNE 2016	FARMLAND	0	385515714	76381	<MDL[1]	231545878900	2324312333	1213335
378	CONTROL MS	JUNE 2016	FARMLAND	0	169868424	0	<MDL[1]	236000000000	13858597	94455
500	CONTROL MS	JUNE 2016	FARMLAND	0	553762431	0	0	125000000	82805	375302
574	CONTROL MS	JUNE 2016	FARMLAND	0	19013101	0	0	491500	0	183313
316	BLUEBERRY MS	JUNE 2016	BLUEBERRY	0	21100566	0	0	3342216	0	97479
361	BLUEBERRY MS	JUNE 2016	BLUEBERRY	0	83004083	0	0	0	0	1034051
469	BLUEBERRY MS	JUNE 2016	BLUEBERRY	0	231587132	0	0	5572401	0	1585049
582	BLUEBERRY MS	JUNE 2016	BLUEBERRY	0	498576369	0	0	704989	18203	3758558
596	BLUEBERRY MS	JUNE 2016	BLUEBERRY	0	348319571	0	0	622455135	<MDL[1]	1924274
485	CRANBERRY MS	JUNE 2016	FARMLAND	0	734319279	8300	0	378500000000	3404199378	564778
492	CRANBERRY MS	JUNE 2016	FARMLAND	0	116166081	0	0	45648189	11827884	44042
573	CRANBERRY MS	JUNE 2016	FARMLAND	0	512904276	0	0	1475000	<MDL[1]	504151
578	CRANBERRY MS	JUNE 2016	FARMLAND	0	323476844	0	0	67000000	22522	5173697
587	CRANBERRY MS	JUNE 2016	FARMLAND	0	43751459	0	0	0	0	508564
200	DOUBLE MS	JUNE 2016	BLUEBERRY	0	1017915931	0	0	67000000	21607891	1720506
488	DOUBLE MS	JUNE 2016	BLUEBERRY	0	530583584	0	0	4770000000	67650	1032953
516	DOUBLE MS	JUNE 2016	BLUEBERRY	0	177044078	0	<MDL[1]	1850000000	108170474	61043811400
546	DOUBLE MS	JUNE 2016	BLUEBERRY	0	1129408967	0	0	8550	0	1097818
598	DOUBLE MS	JUNE 2016	BLUEBERRY	0	1252115045	0	0	397500	25087	83066268200
206	CONTROL MS	JULY 2016	FARMLAND	0	975244670000	0	0	12250000	2762180	349815
221	CONTROL MS	JULY 2016	FARMLAND	0	1369083475	<MDL[1]	<MDL[1]	0	0	1716015445
378	CONTROL MS	JULY 2016	FARMLAND	0	158725339	0	0	1790000000000	5500168180	562928
500	CONTROL MS	JULY 2016	FARMLAND	0	74308211	0	0	0	0	2195615045
574	CONTROL MS	JULY 2016	FARMLAND	0	124650927	0	<MDL[1]	6650	0	72638
316	BLUEBERRY MS	JULY 2016	FARMLAND	0	171115611	0	0	23550	0	87440
361	BLUEBERRY MS	JULY 2016	FARMLAND	0	523849688	0	0	0	0	276232
469	BLUEBERRY MS	JULY 2016	FARMLAND	0	2001876414	0	0	0	0	133188
582	BLUEBERRY MS	JULY 2016	FARMLAND	0	324629576	0	0	272000	0	59494676750
596	BLUEBERRY MS	JULY 2016	FARMLAND	0	970636196	0	0	11750	0	14389452610
485	CRANBERRY MS	JULY 2016	CRANBERRY	0	261721619	0	<MDL[1]	0	0	2553661
492	CRANBERRY MS	JULY 2016	CRANBERRY	0	5635451570	0	0	168500	0	3525284
573	CRANBERRY MS	JULY 2016	CRANBERRY	0	117168835	0	<MDL[1]	11400	0	1785268507
578	CRANBERRY MS	JULY 2016	CRANBERRY	0	766316992500	0	0	8200	0	271082815800
587	CRANBERRY MS	JULY 2016	CRANBERRY	0	321208522	0	0	25700	0	47302007870
200	DOUBLE MS	JULY 2016	CRANBERRY	0	24767474105	0	<MDL[1]	26050	0	17019079320
488	DOUBLE MS	JULY 2016	CRANBERRY	0	231643859	0	<MDL[1]	1390000000	74499877	1033515
516	DOUBLE MS	JULY 2016	CRANBERRY	0	325989058	0	<MDL[1]	3490000	18926	38929319290
546	DOUBLE MS	JULY 2016	CRANBERRY	0	24124774715	0	0	172500000000	84789	1922213
598	DOUBLE MS	JULY 2016	CRANBERRY	0	923989769500	0	0	61000	25054	100042486100
206	CONTROL MS	AUGUST 2016	FARMLAND	0	139658597	<MDL[1]	0	185500	0	204443
221	CONTROL MS	AUGUST 2016	FARMLAND	0	39936634800	735	0	0	0	53955
378	CONTROL MS	AUGUST 2016	FARMLAND	0	1667364	0	0	0	0	1199959
500	CONTROL MS	AUGUST 2016	FARMLAND	<MDL[1]	147250932	23650	0	39350	0	1167270
574	CONTROL MS	AUGUST 2016	FARMLAND	0	657048941	0	0	0	0	92710
316	BLUEBERRY MS	AUGUST 2016	FARMLAND	0	53357211	0	0	14150	0	65103
361	BLUEBERRY MS	AUGUST 2016	FARMLAND	0	4417696686	0	<MDL[1]	0	0	633137
469	BLUEBERRY MS	AUGUST 2016	FARMLAND	0	58716226	0	30550	0	0	79518
582	BLUEBERRY MS	AUGUST 2016	FARMLAND	0	25247381	<MDL[1]	0	0	0	156334
596	BLUEBERRY MS	AUGUST 2016	FARMLAND	0	461409844	0	0	57500	0	1910914311
485	CRANBERRY MS	AUGUST 2016	FARMLAND	0	244373137	0	<MDL[1]	<MDL[1]	0	19211481575
492	CRANBERRY MS	AUGUST 2016	FARMLAND	0	37562747	0	0	11300	<MDL[1]	3675462
573	CRANBERRY MS	AUGUST 2016	FARMLAND	0	10983669210	0	0	935000	<MDL[1]	8743734
578	CRANBERRY MS	AUGUST 2016	FARMLAND	0	22725638170	0	0	44700000	171326	4186130610
587	CRANBERRY MS	AUGUST 2016	FARMLAND	0	15039863560	0	0	0	0	11079420
200	DOUBLE MS	AUGUST 2016	FARMLAND	0	178199290	<MDL[1]	0	0	0	98428612450
488	DOUBLE MS	AUGUST 2016	FARMLAND	0	445469095	0	0	126000	12482	237632
516	DOUBLE MS	AUGUST 2016	FARMLAND	0	201021211	0	0	164000000	5200938	70067844200
546	DOUBLE MS	AUGUST 2016	FARMLAND	0	183811322	0	104500	<MDL[1]	0	441827
598	DOUBLE MS	AUGUST 2016	FARMLAND	0	220893381	0	0	3575	0	93170806550
206	CONTROL MS	MAY 2017	FARMLAND	0	427500000	1085	73000	2685	0	2130
221	CONTROL MS	MAY 2017	FARMLAND	0	6200000000	<MDL[1]	<MDL[1]	0	0	2245
378	CONTROL MS	MAY 2017	FARMLAND	Dead colony						
500	CONTROL MS	MAY 2017	FARMLAND	0	120500000	0	0	0	0	1380
574	CONTROL MS	MAY 2017	FARMLAND	0	232000000	0	<MDL[1]	0	0	2040
316	BLUEBERRY MS	MAY 2017	FARMLAND	0	308500000	0	0	122500	0	213
361	BLUEBERRY MS	MAY 2017	FARMLAND	0	69000000	2070	<MDL[1]	0	0	6050
469	BLUEBERRY MS	MAY 2017	FARMLAND	0	107000000	0	<MDL[1]	6350	0	278
582	BLUEBERRY MS	MAY 2017	FARMLAND	0	328500000	0	0	0	0	180
596	BLUEBERRY MS	MAY 2017	FARMLAND	0	2100000000	0	0	0	0	91500
485	CRANBERRY MS	MAY 2017	FARMLAND	Dead colony						
492	CRANBERRY MS	MAY 2017	FARMLAND	Dead colony						
573	CRANBERRY MS	MAY 2017	FARMLAND	<MDL[1]	1110000000	0	<MDL[1]	0	0	2500000000
578	CRANBERRY MS	MAY 2017	FARMLAND	0	95000000	0	0	0	0	288
587	CRANBERRY MS	MAY 2017	FARMLAND	Dead colony						
200	DOUBLE MS	MAY 2017	FARMLAND	Dead colony						
488	DOUBLE MS	MAY 2017	FARMLAND	0	1275000000	0	0	0	0	900
516	DOUBLE MS	MAY 2017	FARMLAND	0	112000000	0	<MDL[1]	0	0	515000
546	DOUBLE MS	MAY 2017	FARMLAND	0	94000000	<MDL[1]	<MDL[1]	0	0	1540
598	DOUBLE MS	MAY 2017	FARMLAND	0	13700000000	0	0	11050	2055	4040000000

[1] "<MDL" indicates the number of virus copies was below the minimum detection limit but was positive
