# Supplementary material for: The impact of lowbush blueberry (Vaccinium angustifolium Ait.) and cranberry (Vaccinium macrocarpon Ait.) pollination on honey bee (Apis mellifera L.) colony health status
Source: PLoS One. 2020 Jan 24;15(1):e0227970. doi: 10.1371/journal.pone.0227970 (PMC6980599; doi:10.1371/journal.pone.0227970)
Supplement: S5 Table — (PDF) [file pone.0227970.s005.pdf]

BEEHIVE#	MANAGEMENT STRATEGIES	TIME	APIARY	VIRUSES IN FORAGER BEES						
				ABPV	BQCV	CBPV	DWV	IAPV	KBV	SBV
206	CONTROL MS	MAY 2016	FARMLAND	0	2789355065	11137255	<MDL[1]	4481	0	72017
221	CONTROL MS	MAY 2016	FARMLAND	0	5735083310	0	0	117631	<MDL[1]	40098
378	CONTROL MS	MAY 2016	FARMLAND	0	986250330	0	0	491	0	599
500	CONTROL MS	MAY 2016	FARMLAND	0	1856036433	<MDL[1]	0	3463	0	312
574	CONTROL MS	MAY 2016	FARMLAND	0	653853097	0	0	0	0	0
316	BLUEBERRY MS	MAY 2016	FARMLAND	0	1772457739	4630	0	1172058	0	15020
361	BLUEBERRY MS	MAY 2016	FARMLAND	0	9346916310	<MDL[1]	0	15256	<MDL[1]	9990
469	BLUEBERRY MS	MAY 2016	FARMLAND	0	7203109875	0	0	50504	<MDL[1]	7750
582	BLUEBERRY MS	MAY 2016	FARMLAND	0	629494437	0	0	0	0	8015
596	BLUEBERRY MS	MAY 2016	FARMLAND	0	1823274771	824	0	342552366200	45744	<MDL1
485	CRANBERRY MS	MAY 2016	FARMLAND	0	561732851	0	0	0	0	1348
492	CRANBERRY MS	MAY 2016	FARMLAND	0	29488286920	0	0	0	<MDL[1]	425
573	CRANBERRY MS	MAY 2016	FARMLAND	0	9488913120	0	0	0	0	183
578	CRANBERRY MS	MAY 2016	FARMLAND	0	925991543	154067	0	0	0	32211
587	CRANBERRY MS	MAY 2016	FARMLAND	0	594146076	0	0	0	0	2145
200	DOUBLE MS	MAY 2016	FARMLAND	0	1111245238	0	<MDL[1]	0	0	<MDL1
488	DOUBLE MS	MAY 2016	FARMLAND	0	4684792057	<MDL[1]	<MDL[1]	26012485	122593	2141
516	DOUBLE MS	MAY 2016	FARMLAND	0	1244504362	0	0	4746	0	15763881
546	DOUBLE MS	MAY 2016	FARMLAND	0	6240281590	<MDL[1]	0	1247661	93175	203077
598	DOUBLE MS	MAY 2016	FARMLAND	0	326605715	494	0	0	0	15102
206	CONTROL MS	JUNE 2016	FARMLAND	0	46700289595	0	0	4117287	3285103	22731
221	CONTROL MS	JUNE 2016	FARMLAND	0	1376226451	0	0	4121	0	111901921
378	CONTROL MS	JUNE 2016	FARMLAND	0	208250483	0	<MDL[1]	1890207910000	18332374870	70755
500	CONTROL MS	JUNE 2016	FARMLAND	0	68242626	0	0	1524	0	5298
574	CONTROL MS	JUNE 2016	FARMLAND	0	938564492	0	0	62544664	181702	7879
316	BLUEBERRY MS	JUNE 2016	BLUEBERRY	0	96292380800	0	0	50531	26090	5626130335
361	BLUEBERRY MS	JUNE 2016	BLUEBERRY	0	16578490115	0	0	3063997675	362183	439279
469	BLUEBERRY MS	JUNE 2016	BLUEBERRY	0	926766445	<MDL[1]	0	795940297	0	1806652
582	BLUEBERRY MS	JUNE 2016	BLUEBERRY	0	770791295	0	0	0	0	4176885
596	BLUEBERRY MS	JUNE 2016	BLUEBERRY	0	41040238850	0	0	8053	0	117646
485	CRANBERRY MS	JUNE 2016	FARMLAND	0	155495618	0	0	1187371	3191	265754
492	CRANBERRY MS	JUNE 2016	FARMLAND	0	18590388760	0	0	1623797	41442	28254
573	CRANBERRY MS	JUNE 2016	FARMLAND	0	201061078	0	0	8706479	<MDL[1]	151303
578	CRANBERRY MS	JUNE 2016	FARMLAND	0	208170240	0	0	0	0	6584
587	CRANBERRY MS	JUNE 2016	FARMLAND	0	17732988260	5291	35175	1298635649000	2591852	644823
200	DOUBLE MS	JUNE 2016	BLUEBERRY	0	12513475710	0	0	304793	24308	73935
488	DOUBLE MS	JUNE 2016	BLUEBERRY	0	2776136299	0	0	48528215300	100664198	61376
516	DOUBLE MS	JUNE 2016	BLUEBERRY	0	633318139	0	<MDL[1]	10193993	1994448	1718395176
546	DOUBLE MS	JUNE 2016	BLUEBERRY	0	83380279300	0	<MDL[1]	83361967	<MDL[1]	873575
598	DOUBLE MS	JUNE 2016	BLUEBERRY	0	55096907700	0	0	15098	0	25329876265
206	CONTROL MS	JULY 2016	FARMLAND	0	15045583080	84688	0	618543444	59643153	6845998480
221	CONTROL MS	JULY 2016	FARMLAND	400	13719527945	526074	0	3233387266	1664889411	3319053
378	CONTROL MS	JULY 2016	FARMLAND	0	301522312	13062	0	259793510	1289428	6654574
500	CONTROL MS	JULY 2016	FARMLAND	0	260092067	22165	0	6658	0	19365
574	CONTROL MS	JULY 2016	FARMLAND	0	1331334731	0	<MDL[1]	71052814200	1348448732	5684
316	BLUEBERRY MS	JULY 2016	FARMLAND	0	164988925650	4274	0	8008	0	22228
361	BLUEBERRY MS	JULY 2016	FARMLAND	0	499312312	<MDL[1]	0	0	0	873853
469	BLUEBERRY MS	JULY 2016	FARMLAND	0	7385646430	42722	0	0	0	161060
582	BLUEBERRY MS	JULY 2016	FARMLAND	<MDL[1]	3092231958	420	0	0	0	4322444
596	BLUEBERRY MS	JULY 2016	FARMLAND	0	4137020162	0	0	<MDL[1]	0	885977528
485	CRANBERRY MS	JULY 2016	CRANBERRY	0	46642633705	0	0	4439	0	117255
492	CRANBERRY MS	JULY 2016	CRANBERRY	0	852954682	<MDL[1]	0	11021	0	1211695
573	CRANBERRY MS	JULY 2016	CRANBERRY	0	259067364	0	31535	0	0	510167
578	CRANBERRY MS	JULY 2016	CRANBERRY	0	36942752030	<MDL[1]	0	<MDL[1]	<MDL[1]	690924
587	CRANBERRY MS	JULY 2016	CRANBERRY	0	1190248448	0	0	652295622	7404	670243
200	DOUBLE MS	JULY 2016	CRANBERRY	0	1135435127	367	0	119463	0	169692
488	DOUBLE MS	JULY 2016	CRANBERRY	0	520540455	0	0	53980	9948	548006
516	DOUBLE MS	JULY 2016	CRANBERRY	0	18130271535	17709	0	3709119	324729	383907
546	DOUBLE MS	JULY 2016	CRANBERRY	0	3625412469	0	0	54902	<MDL[1]	10031074
598	DOUBLE MS	JULY 2016	CRANBERRY	0	82537365900	32388	11394784	40859	<MDL[1]	22581478940
206	CONTROL MS	AUGUST 2016	FARMLAND	0	197622962	0	0	3586063046	340744989	1321272
221	CONTROL MS	AUGUST 2016	FARMLAND	0	820332380	4027420	0	0	0	5731890
378	CONTROL MS	AUGUST 2016	FARMLAND	0	21230396	873520	0	<MDL[1]	0	8619
500	CONTROL MS	AUGUST 2016	FARMLAND	0	24694670	<MDL[1]	0	0	0	12505
574	CONTROL MS	AUGUST 2016	FARMLAND	0	121325589	0	0	0	0	10346
316	BLUEBERRY MS	AUGUST 2016	FARMLAND	0	861214104000	0	0	0	0	6081671585
361	BLUEBERRY MS	AUGUST 2016	FARMLAND	0	45222804	0	<MDL[1]	857733	0	653504141
469	BLUEBERRY MS	AUGUST 2016	FARMLAND	0	128014211	49044	0	<MDL[1]	0	193702
582	BLUEBERRY MS	AUGUST 2016	FARMLAND	0	71926343750	0	0	0	0	2289737221
596	BLUEBERRY MS	AUGUST 2016	FARMLAND	0	1388502122	0	0	0	0	936725007
485	CRANBERRY MS	AUGUST 2016	FARMLAND	0	73151279	<MDL[1]	0	0	0	2351946
492	CRANBERRY MS	AUGUST 2016	FARMLAND	0	159989770	0	0	1249765868	21021552	17006525620
573	CRANBERRY MS	AUGUST 2016	FARMLAND	0	586163604	1023	0	64152	0	607135
578	CRANBERRY MS	AUGUST 2016	FARMLAND	0	49076088250	2392785	0	4956639	<MDL[1]	13220360140
587	CRANBERRY MS	AUGUST 2016	FARMLAND	0	30432286590	497099077	0	16065	0	1451985
200	DOUBLE MS	AUGUST 2016	FARMLAND	0	383470903	<MDL[1]	0	5299	0	44963
488	DOUBLE MS	AUGUST 2016	FARMLAND	0	<MDL[1]	<MDL[1]	0	135361	0	<MDL1
516	DOUBLE MS	AUGUST 2016	FARMLAND	0	292383700800	0	0	20463743335000	2433100127	2732474849
546	DOUBLE MS	AUGUST 2016	FARMLAND	0	152132071	<MDL[1]	0	3978	0	37231
598	DOUBLE MS	AUGUST 2016	FARMLAND	0	23926294480	62674	<MDL[1]	2968414959	0	389375975150
206	CONTROL MS	MAY 2017	FARMLAND	0	8000000000	192	0	0	0	5550
221	CONTROL MS	MAY 2017	FARMLAND	<MDL[1]	4430000000000	0	<MDL[1]	0	0	2575000
378	CONTROL MS	MAY 2017	FARMLAND	Dead colony						
500	CONTROL MS	MAY 2017	FARMLAND	0	263000000000	<MDL[1]	0	0	0	44450
574	CONTROL MS	MAY 2017	FARMLAND	0	520000000	0	0	0	0	1045000
316	BLUEBERRY MS	MAY 2017	FARMLAND	0	266000000	17050000000	0	41450	0	2110
361	BLUEBERRY MS	MAY 2017	FARMLAND	0	1130000000	<MDL[1]	0	0	0	76000
469	BLUEBERRY MS	MAY 2017	FARMLAND	0	273000000	0	0	0	0	197
582	BLUEBERRY MS	MAY 2017	FARMLAND	0	1970000000000	63500	0	5850	0	0
596	BLUEBERRY MS	MAY 2017	FARMLAND	0	2420000000	0	<MDL[1]	0	0	725
485	CRANBERRY MS	MAY 2017	FARMLAND	Dead colony						
492	CRANBERRY MS	MAY 2017	FARMLAND	Dead colony						
573	CRANBERRY MS	MAY 2017	FARMLAND	<MDL[1]	20600000000	0	<MDL[1]	0	<MDL[1]	183500
578	CRANBERRY MS	MAY 2017	FARMLAND	0	47800000	1030	0	0	0	0
587	CRANBERRY MS	MAY 2017	FARMLAND	Dead colony						
200	DOUBLE MS	MAY 2017	FARMLAND	Dead colony						
488	DOUBLE MS	MAY 2017	FARMLAND	0	7350000000	0	0	0	0	0
516	DOUBLE MS	MAY 2017	FARMLAND	0	227500000	0	0	0	0	118
546	DOUBLE MS	MAY 2017	FARMLAND	0	213500000	0	19100	0	0	0
598	DOUBLE MS	MAY 2017	FARMLAND	0	1285000000	530	0	<MDL[1]	0	218000

[1] "<MDL" indicates the number of virus copies was below the <MDLimum detection limit but was positive
