# Supplementary material for: The impact of lowbush blueberry (Vaccinium angustifolium Ait.) and cranberry (Vaccinium macrocarpon Ait.) pollination on honey bee (Apis mellifera L.) colony health status
Source: PLoS One. 2020 Jan 24;15(1):e0227970. doi: 10.1371/journal.pone.0227970 (PMC6980599; doi:10.1371/journal.pone.0227970)
Supplement: S6 Table — (PDF) [file pone.0227970.s006.pdf]

Beehive #	Method / Date / MS	A Alcohol washing June 1 2016 (mite/100 bees)	B Natural mite fall October 27 2016 (mite/day)	C Natural mite fall December 6 2016 (mite/day)	D Natural mite fall June 1 2017 (mite/day)
		Varroa counting			
**206**	**Control MS**	**0.0**	**1.0**	**1.8**	**0.6**
**221**	**Control MS**	**No data**[2]	**1.6**	**0.3**	**0.0**
**378**	**Control MS**	**0.0**	**3.3**	**3.0**	**Dead**[1]
**500**	**Control MS**	**0.0**	**0.0**	**0.8**	**0.0**
**574**	**Control MS**	**0.0**	**0.4**	**0.3**	**0.0**
**316**	**Blueberry MS**	**0.0**	**4.4**	**6.8**	**0.1**
**361**	**Blueberry MS**	**No data**[2]	**0.1**	**0.2**	**0.3**
**469**	**Blueberry MS**	**0.0**	**1.7**	**0.3**	**0.1**
**582**	**Blueberry MS**	**0.0**	**0.4**	**0.0**	**0.0**
**596**	**Blueberry MS**	**0.0**	**0.1**	**0.0**	**0.3**
**485**	**Cranberry MS**	**No data**[2]	**2.0**	**1.7**	**Dead**[1]
**492**	**Cranberry MS**	**No data**[2]	**0.3**	**0.3**	**Dead**[1]
**573**	**Cranberry MS**	**0.0**	**0.6**	**1.0**	**0.3**
**578**	**Cranberry MS**	**0.0**	**0.4**	**0.3**	**0.4**
**587**	**Cranberry MS**	**0.0**	**Dead**[1]	**Dead**[1]	**Dead**[1]
**200**	**Double MS**	**0.0**	**1.3**	**1.3**	**Dead**[1]
**488**	**Double MS**	**0.0**	**1.0**	**1.8**	**0.1**
**516**	**Double MS**	**0.0**	**0.3**	**0.2**	**0.1**
**546**	**Double MS**	**0.0**	**1.1**	**2.5**	**0.3**
**598**	**Double MS**	**0.0**	**0.7**	**0.8**	**0.6**

[1] "Dead" indicates the colony has died over the winter 2016-2017

[2] "No data" indicates the varroa mite counting was not successful
