# Supplementary material for: The impact of lowbush blueberry (Vaccinium angustifolium Ait.) and cranberry (Vaccinium macrocarpon Ait.) pollination on honey bee (Apis mellifera L.) colony health status
Source: PLoS One. 2020 Jan 24;15(1):e0227970. doi: 10.1371/journal.pone.0227970 (PMC6980599; doi:10.1371/journal.pone.0227970)
Supplement: Attachment — Submitted filename: PONE-D-19-18647_Rebuttal letter-Response to reviewers.docx [file pone.0227970.s011.docx]

****Responses are in red under editor or reviewer’s comments and line numbers (in green) we are referring to are the ones in the final document “Manuscript”. Lines indicated correspond to those of the file “Revised manuscript with tracking changes”.**

Journal Requirements:

1. When submitting your revision, we need you to address these additional requirements.

Please ensure that your manuscript meets PLOS ONE's style requirements, including those for file naming. The PLOS ONE style templates can be found at
<http://www.journals.plos.org/plosone/s/file?id=wjVg/PLOSOne_formatting_sample_main_body.pdf> and <http://www.journals.plos.org/plosone/s/file?id=ba62/PLOSOne_formatting_sample_title_authors_affiliations.pdf>

Done

2.  Please include captions for your Supporting Information files at the end of your manuscript, and update any in-text citations to match accordingly. Please see our Supporting Information guidelines for more information: <http://journals.plos.org/plosone/s/supporting-information>.

Now included for Supporting Tables 1 to 6.

3. In your Methods section, please provide additional information regarding the permits you obtained for the work. Please ensure you have included the full name of the authority that approved the field site access and, if no permits were required, a brief statement explaining why.

No permits were required for this research. Colonies where owned by our research center (Centre de recherche en sciences animales de Deschambault) and verbal agreements were taken with the landowners of the various apiaries.

4. Thank you for stating the following in the Financial Disclosure section:

PG received research grant from Bayer Crop Science. Bayer Crop Science participated in the initial study design but was not involved in the final study desing nor the data collection, data anlysis, decision to publish and preparation of manuscript.

Now included in the cover letter.

We note that you received funding from a commercial source: Bayer Crop Science.

Please provide an amended Competing Interests Statement that explicitly states this commercial funder, along with any other [relevant declarations](http://www.plosone.org/static/competing.action) relating to employment, consultancy, patents, products in development, marketed products, etc.

Within this Competing Interests Statement, please confirm that this does not alter your adherence to all PLOS ONE policies on sharing data and materials by including the following statement: "This does not alter our adherence to PLOS ONE policies on sharing data and materials.” (as detailed online in our guide for authors <http://journals.plos.org/plosone/s/competing-interests>).  If there are restrictions on sharing of data and/or materials, please state these. Please note that we cannot proceed with consideration of your article until this information has been declared.

Please include your amended Competing Interests Statement within your cover letter. We will change the online submission form on your behalf.

Please know it is PLOS ONE policy for corresponding authors to declare, on behalf of all authors, all potential competing interests for the purposes of transparency. PLOS defines a competing interest as anything that interferes with, or could reasonably be perceived as interfering with, the full and objective presentation, peer review, editorial decision-making, or publication of research or non-research articles submitted to one of the journals. Competing interests can be financial or non-financial, professional, or personal. Competing interests can arise in relationship to an organization or another person. Please follow this link to our website for more details on competing interests: <http://journals.plos.org/plosone/s/competing-interests>

We added this sentence in the cover letter: “There is no competing interest with regards with this research and authors adhere to PLOS ONE policies on sharing data and materials.”

5.  We note that Figure 1 in your submission contain map/satellite images which may be copyrighted. All PLOS content is published under the Creative Commons Attribution License (CC BY 4.0), which means that the manuscript, images, and Supporting Information files will be freely available online, and any third party is permitted to access, download, copy, distribute, and use these materials in any way, even commercially, with proper attribution. For these reasons, we cannot publish previously copyrighted maps or satellite images created using proprietary data, such as Google software (Google Maps, Street View, and Earth). For more information, see our copyright guidelines: <http://journals.plos.org/plosone/s/licenses-and-copyright>.

 We require you to either (1) present written permission from the copyright holder to publish these figures specifically under the CC BY 4.0 license, or (2) remove the figures from your submission:

1. You may seek permission from the original copyright holder of Figure 1 to publish the content specifically under the CC BY 4.0 license.

 We recommend that you contact the original copyright holder with the Content Permission Form (<http://journals.plos.org/plosone/s/file?id=7c09/content-permission-form.pdf>) and the following text:

“I request permission for the open-access journal PLOS ONE to publish XXX under the Creative Commons Attribution License (CCAL) CC BY 4.0 (<http://creativecommons.org/licenses/by/4.0/>). Please be aware that this license allows unrestricted use and distribution, even commercially, by third parties. Please reply and provide explicit written permission to publish XXX under a CC BY license and complete the attached form.”

 Please upload the completed Content Permission Form or other proof of granted permissions as an "Other" file with your submission.

 In the figure caption of the copyrighted figure, please include the following text: “Reprinted from [ref] under a CC BY license, with permission from [name of publisher], original copyright [original copyright year].”

2. If you are unable to obtain permission from the original copyright holder to publish these figures under the CC BY 4.0 license or if the copyright holder’s requirements are incompatible with the CC BY 4.0 license, please either i) remove the figure or ii) supply a replacement figure that complies with the CC BY 4.0 license. Please check copyright information on all replacement figures and update the figure caption with source information. If applicable, please specify in the figure caption text when a figure is similar but not identical to the original image and is therefore for illustrative purposes only.The following resources for replacing copyrighted map figures may be helpful:

 USGS National Map Viewer (public domain): <http://viewer.nationalmap.gov/viewer/>

The Gateway to Astronaut Photography of Earth (public domain): <http://eol.jsc.nasa.gov/sseop/clickmap/>

Maps at the CIA (public domain): <https://www.cia.gov/library/publications/the-world-factbook/index.html> and <https://www.cia.gov/library/publications/cia-maps-publications/index.html>

NASA Earth Observatory (public domain): [http://earthobservatory.nasa.gov/](http://earthobservatory.nasa.gov/%20)

Landsat: <http://landsat.visibleearth.nasa.gov/>

USGS EROS (Earth Resources Observatory and Science (EROS) Center) (public domain): [http://eros.usgs.gov/#](http://eros.usgs.gov/)

Natural Earth (public domain): <http://www.naturalearthdata.com/>

It is not a Google map but an ArcGIS Map by ESRI. After verifications, the following citation (added in caption of Fig 1 and Fig 3) will meet PLoS ONE requirements:

*Sources: Esri, DigitalGlobe, GeoEye, i-cubed, USDA FSA, USGS, AEX, Getmapping, Aerogrid, IGN, IGP, swisstopo, and the GIS User Community 'Maps throughout this book were created using ArcGIS® software by Esri. ArcGIS® and ArcMap™ are the intellectual property of Esri and are used herein under license. Copyright © Esri. All rights reserved. For more information about Esri® software, please visit www.esri.com.'*

Additional Editor Comments:

I agree with both reviewers that this paper is interesting but requires some major revisions to improve clarity. In particular, the claims made in the discussion may be valid, but it is hard for the reader to verify this because the methodological details are limited or confusing. See Reviewer 1's comprehensive comments about this.

The description of the study design set up is quite confusing - it would help the read if the authors could specifically describe how many colonies were under which treatment, and at what times. For example, in Table 1 the authors could include (n = ?) in each box to show how many of the 20 colonies were in each treatment at a given stage of the experiment.

We agree and Table 1 has been modified accordingly.

In addition, the statistical analysis requires some more clarification. I also found the description of the modelling approach confusing (lines 235-259). I agree that each colony is an experimental unit, but because some colonies are moved, there is a 'time X location' interaction here - it's not clear if this was taken into consideration. Also see Reviewer 2's comment about addressing pseudoreplication. The details of the mixed models are not given - this needs to be more clear. Should the mixed models include colony number as a random effect to account for movement issue?

We used a two-way Anova model with repeated measurements to analyze the data. This model considers the treatment as the between-subject factor, the time as the within-subject factor, and the interaction treatment*time. All previous factors are fixed in the model, while colonies nested in treatment are considered as a random factor. We have clarified the description of the model in the methodology. Line 255 to 293.

The interaction treatment*time is already present in the model. This interaction allows us to compare the treatment profiles (control management strategy / blueberry management strategy / cranberry management strategy / double management strategy) over time.


[Note: HTML markup is below. Please do not edit.]

Reviewers' comments:

Reviewer's Responses to Questions

**Comments to the Author**

1. Is the manuscript technically sound, and do the data support the conclusions?

The manuscript must describe a technically sound piece of scientific research with data that supports the conclusions. Experiments must have been conducted rigorously, with appropriate controls, replication, and sample sizes. The conclusions must be drawn appropriately based on the data presented. 

Reviewer #1: No

Reviewer #2: Yes

2. Has the statistical analysis been performed appropriately and rigorously? 

Reviewer #1: I Don't Know

Reviewer #2: Yes

3. Have the authors made all data underlying the findings in their manuscript fully available?

The [PLOS Data policy](http://www.plosone.org/static/policies.action#sharing) requires authors to make all data underlying the findings described in their manuscript fully available without restriction, with rare exception (please refer to the Data Availability Statement in the manuscript PDF file). The data should be provided as part of the manuscript or its supporting information, or deposited to a public repository. For example, in addition to summary statistics, the data points behind means, medians and variance measures should be available. If there are restrictions on publicly sharing data—e.g. participant privacy or use of data from a third party—those must be specified.

Reviewer #1: No

Reviewer #2: Yes

4. Is the manuscript presented in an intelligible fashion and written in standard English?

PLOS ONE does not copyedit accepted manuscripts, so the language in submitted articles must be clear, correct, and unambiguous. Any typographical or grammatical errors should be corrected at revision, so please note any specific errors here.

Reviewer #1: No

Reviewer #2: Yes

5. Review Comments to the Author

Please use the space provided to explain your answers to the questions above. You may also include additional comments for the author, including concerns about dual publication, research ethics, or publication ethics. (Please upload your review as an attachment if it exceeds 20,000 characters)

Reviewer #1: This study aimed to determine whether using honey bee colonies for commercial pollination of cranberries, blueberries or both resulted in negative impacts on their health compared to colonies used for honey production only. They hypothesised that the bees exposed to more diverse and abundant floral resources ie the honey production colonies, would be healthier (as assessed by measures of colony development and parasite and pathogen loads) than their counterparts exposed to a reduced floral diversity (monoculture crops). Their key finding was that bees used for commercialisation of cranberry (both cranberry only and cranberry + blueberry) experiences significantly less weight gain compared to control and blueberry only bees. They also saw differences in capped brood area, virus loads and Nosema levels (blueberry only) during pollination seasons.
While I think this experimental setup is sound and the results are novel and interesting, the conclusions claim far more than can reasonably be inferred from the results, some of which do not appear to be reported at all or are there but not described in sufficient detail. Furthermore, the writing is often unclear or does not flow well.

General points:

1. While the difference in weight gain appears to be the most striking result, I believe too much is concluded from this. While this result is consistent with the capped brood data during June and July, by August and May2017 the brood area had returned to comparable levels which suggests to me the differences were largely due to honey stores which is not surprising given that the honey producing colonies were presumably put into an area with a particularly good honey flow which the authors state in the discussion was during cranberry pollination period.

During cranberry pollination, colonies in cranberry MS and double MS suffered from low nectar flow while at the same time colonies in control MS and Blueberry MS were in the farmland apiary with a good honey flow. We added a sentence about the brood population in relation to the different MS. Line 362 to 365.

2. This manuscript discussed negative effects of pollination on bee health but it seems to me that all colonies remained largely healthy. The paper discusses colony mortality but nowhere is this data shown so I think it is difficult to infer any ‘carry over’ effects when it seems all colonies made it through winter with little, if any difference to any of the metrics assessed.

We agree, colony mortality information was missing, it is now included. Line 450 to 452 and line 517 to 520.

3. I feel this manuscript would benefit from an overall rewrite to improve clarity and flow. See specific points for some examples.

Specific points:
L31. Sacbrood is one word

It has been corrected. Line 36, 202, 405.

L36. You list three things here which are quite vague and overlapping without going into what you mean. You only give the example of increasing monoculture crops. It would be better if you gave further examples of what you mean, or stick to just discussing monocultures.

We agree this was irrelevant information and we removed it.

L46-50. This paragraph doesn’t flow smoothly, there are a number of facts and numbers but reported quite differently and it is confusing to read eg blueberry fruit set rises from 30% to 100% and Carberry increases by 50% but from what to what? It reads like something is missing when you have included that info for blueberries.

We agree. The sentence has been modified and we removed the yield information that was confusing. Line 48-50.

L52. Add (Canada) at the first mention of Quebec

It has been corrected. Line 51.

L52-58. This paragraph, along with a number of others throughout the manuscript, including the previous one, to me does not flow well and instead just reads like a list of stats. I think this could benefit from some re-wording to improve the flow.

We have made many changes in the manuscript that help the "flow" of information.

L60. Change ‘has’ to ‘can have’ etc. Please go through this paragraph to tone down statements. I don’t think previous research has been conclusive enough that using bees for pollination of monocultures always has a detrimental effect on their health, hence why your study is interesting.

We agree and we made changes in this paragraph. Lines 60, 64, 65, 66, 76, 77.

L76-77. This sentence is confusing, do you mean in the wider environment compared to the crop or generally bees need pollen and nectar? If the first is what you mean, I think this is commonly untrue, if the latter, it seems an unnecessary sentence in a strange place.

We agree this is confusing. We removed it.

L91. Specify you mean a negative impact

We added the word “negative”. Line 89.

L93-96. I don’t quite understand this? Do you mean this manuscript is the first part of a two-part study? This seems unusual to me but I don’t know what the journal’s guidelines are with these matters.

We agree that this was confusing. We deleted this information.

L107. This is the first mention of a farmland apiary and you don’t seem to use the term again. I think perhaps it could be worded differently as blueberry and cranberry farm are also farmland. Perhaps state what kind of farm it is?

The term “Farmland apiary” is used throughout the manuscript. But we agree additional information would help to understand what it is exactly. Line 105-106.

L112. You don’t need ‘was carefully selected’

We agree that only “was selected” is enough. Line 111.

L123. By ‘from the dates presented earlier’ do you mean May 2016?

This confusion has been eliminated by referring to Table 1 instead. Line 127.

L124. This would read better “for blueberries (5 colonies),cranberries (5 colonies) or both (5 colonies)”

We agree. Line 125 to 129.

L132. Is “Propolis-etc..” the name of the manufacturer? If so please also give the location.

This is indeed the case. We added the location. Line 136.

L140. This table would be clearer with dates across the top and location by colour, as with Table 3

We agree and we modified the Table 1. Line 145.

Please note Table 3 has been removed (more details below).

L191-229. Were the different life stages analysed separately or together? How many of each life stage were analysed? How were individuals pooled? 280 individuals is a lot to be collected to only analyse 10, was there a particular reason for this? I’m not familiar with the pathogen RNA/DNA kit, does it somehow enrich your samples? Can you explain your qPCR controls/standards? How did you calculate per reaction copy number? Did using neat RNA/DNA yield quantifiable results for all samples without the need to dilute any? Etc etc ....more info is needed in this section.

We added more information in the text. Line 202-209 and 222-224.

Here are some specific answers to your questions: Extra bees were submitted in case repeat testing was required, and also allowed for the excess to be stored for future analysis if desired. The MagMAX Pathogen RNA/DNA kit (Applied Biosystems) is the set of reagents used by the MagMAX automated nucleic extraction platform to extract the nucleic acid from the bee pools. Positive controls for the qPCRs are in the form of synthetic DNA constructs (gBlocks, IDT) containing the target sequence for the individual assays. A previously-generated standard curve for each assay using serial dilutions of these controls was used as an external standard curve for calculating sample concentration, as each qPCR assay included four serial dilutions (E8 through E5 copies per uL) of its corresponding gBlock as in-run standards. The gBlocks were also used as positive extraction controls. Negative extraction and negative template controls were also used. An assay was considered to have passed QC if the negative controls were negative, and if the positive controls yielded Ct values and Tm curves within the predicted range based on prior experiments. Per-reaction copy numbers were obtained using the external standard curve plus in-run standards described above. Dilution was not required for any samples.

L230. When were Varroa drops recorded? They aren’t mentioned in your sampling plan.

We agree it was missing and we added it. Line 237-243.

L237. I’m afraid I don’t know enough about these analyses to comment on this aspect of the manuscript.

L270. Why do you only discuss composition of the good foraging landscape at 5km radii? What about at 1km and 3km. Maybe this information should be presented in a supplementary table.

We agree. A supplementary table and text were added. Line 299-302.

L302. Specify between all groups

We added the word “all”. Line 338.

L307. These dates are confusing and don’t seem to align with the sampling schedule.

Please see M&M section: "Hives were weighed individually throughout the season each time a honey super was added or removed." Line 170-171. We changed nothing here.

L324. Please state ‘compared to control and cranberry’ and clarify that it was only at the first date of sampling in blueberry pollination, you don’t have the data to compare at the second date. Also the pollen weight did increase in the hives that were in blueberry farms from the first to second sampling dates. So perhaps more blueberry pollen was available/collected in the later part of the flowering? I think you need to be more cautious in your reporting of this data. Also, unless I am misunderstanding your data, there were other significant results eg before pollination the double ms had collected less pollen than the control. Please explain the missing data.

We agree. After considerations, these results do not add relevant information and all data about pollen weight have been removed from the manuscript.

L345. It can also be seen that the amount of capped brood returned to comparable amounts between all treatments in August and remained the same in the following spring. You should be careful to report results in an unbiased way and ensure selectively mentioning some parts and not others isn’t leading to making unsupported conclusions.

We agree. We rewrote results and discussion about capped brood. Line 362-365 and Line 503-506.

L356. You don’t mention queen loss in the methods or give the actual results. You need to provide more information on what you saw. Grouping swarming and queen supersedure and loss all together is not necessarily appropriate as they can be due to vastly differently factors. Ie swarming can be due to a successful colony outgrowing the hive boxes, or supersedure may follow the colony’s removal of a queen that is not performing.

We agree. We changed the text in order to add some clarifications. Line 247-251 and line 445 to 448.

L361-382. This section is quite unclear to me ie:
• it mentions forager bees in particular but doesn’t report any results differentiated by caste
• If “ABPV was in one … sample only” then “there was no significant difference between groups” seems an odd sentence?
• Stating that some viruses were “rarely detected” in particular castes isn’t enough and the results should be reported, summarised in a supplementary table perhaps?
• “The most important infection” needs rewording
• Etc. Please revisit this section and clarify.

We agree it is unclear. This section was completely revised and we added supplementary tables (S2 Table, S3 Table, S4 Table, S5 Table). Line 377-426.

Tables 4 and 5: personally I find this information hard to read in a table and would prefer graphs or a table but with grey-scaling according to copy number. Please specify why Spring 2017 SBV data is missing.

We agree and we changed Tables 4 and 5 for figures instead (Fig 6 and Fig 7). Line 397 and 405.

L408. The methods stated that no differentiation was made between N. apis and N. ceranae so how do you know it is N. ceranae?

We agree this is an error. We did differentiate *N. apis* and *N. ceranae* in the analysis. Line 233-236

L417. Why do you multiple the spores/bee by 10^6?

We agree and removed it. Line 397 and line 405.

L424. I would like to see the actual mite drop data in the supp.

We added a supporting table (S6 Table). Line 444.

L438-444. I believe these conclusion and confirmations of other groups’ findings to be largely overblown.eg “as long as the vegetation in the vicinity is protected and left in a natural state” wasn’t something you tested. You didn’t assess the attractiveness of particular crops/floral resources int his study.

We agree and we removed that information..

L445-448. are unnecessary

We agree and we removed them.

L449-455. This seems odd her. You didn’t assess attractiveness nor pollination/crop yield.

We agree and we removed it.

L461. What are these observations?

All data, results and discussion about pollen weight have been removed as we now believe it was confusing and did not add anything relevant to the study.

L463. If you suspect the pollen traps did not work consistently for colonies in blueberries, it suggests you think the data from this point is an underestimate. If so exercise caution with your conclusion that bees were able to collect less pollen from blueberries.

All data, results and discussion about pollen weight has been removed as we now believe it was confusing and did not add anything relevant to the study.

L465-466. Why do you assume this?

All data, results and discussion about pollen weight has been removed as we now believe it was confusing and did not add anything relevant to the study.

L473-474. You cannot confirm this from your data

All data, results and discussion about pollen weight has been removed as we now believe it was confusing and did not add anything relevant to the study.

L481. What does “critical decline” mean in this case?

We agree “critical” is too conclusive and we removed it. Line 470.

L496. Did it rain at all sites? Perhaps if the bees could not fly and forage this is the reason for the weight gain differences at this period?

We agree this information was missing and we added it (Line 485) We also suggest this is part of the reason for the weight gain differences at this period. Line 486-488.

L516. You have winter mortality but nowhere is this mentioned in methods or results.

We agree and as mentioned above, it is now included. Line 252-254 and line 449-452 and line 518-520.

L529. This is misleading, some differences were seen prior to pollination too, and afterwards results differences disappeared.

We agree. We made changes in the result section for virology (378-426), as well as in the discussion (line 522 to 532).

L539-540. This is inaccurate, or you have not shown important results.

We agree and we removed this affirmation. Removed from actual 538.

L547. See Welch et al 2009, Glenny et al 2017

Very interesting and now included in the discussion. Line 547-550 and line 553-555.

L563-565. I think your wording of the research question needs changing

We agree it needed rewording. Line 566-568.

L573-576. Was this data published previously? Is there a reference?

This is unpublished data. Line 577-578.

Reviewer #2: Overall, I thought this was an interesting study and is well-written. Below are a few edits/comments that I think will potentially make the manuscript stronger.

Line 16- change to "...crops benefit from the presence of honey bees..."

We agree and we made the change. Line 19.

Lines 66-70- I found this section a bit hard to read. I think the authors could re-word this to make it flow better.

We agree and we rewrote this section. Line 59-75.

Lines 93-96- I do not think this is needed and it should be deleted.

We agree and removed it. Before actual line 92.


Line 152- change to "Data were..." Data is plural and I think the authors made this mistake a few times within the manuscript. See line 154- "...Quebec and were analysed..."
Additionally see lines 170 and 175.... Data were (or are)…

We made the changes. Line 155 and line 173.

Line 245- I do not understand how changing the alpha level limits pseudoreplication. You either have or have not pseudoreplication within your study...regardless of the alpha level. Perhaps clarify this within the manuscript.

Some experimental units (colonies) are moving over time, but remained on the same blueberry or cranberry field. This is a lack in the experiment, since colonies should have been moved to different blueberry or cranberry fields. Colonies are thus a form of pseudoreplication and inference is valid only on the specific fields used in the experiment. We have tried to say something about what could be observed on some other blueberry and cranberry fields, by assuming that the observed between colonies variability should be smaller than the one observed when colonies are moved to different fields, leading thus to liberal tests. This is why we used the 0.01 level of significance instead of 0.05.

Line 357- This occurred in 9 of 20 colonies? This seems like a lot? I'm confused why you would have had numerous colonies swarm from the cranberry MS if the colonies were not obtaining enough resources and were decreasing in weight. I would assume that swarming would have been rare in this treatment?

The swarming occurred in June, in the MS colonies that were in the farmland apiary where resources were abundant and led to higher rate of colony development. The section has been rewritten. Line 445-448.

Line 420- change to (HSD)

We presume you mean removing the second parenthesis? We made changes. Line 356, 374, 401, 409 and 439.


Line 446- I think this statement needs a citation. And by "wild" do you mean feral honey bee colonies or native bees in general?

We agree that this is confusing. We removed it. Before line 467.

Lines 584-587- If you feed colonies substantive quantities of pollen and/or sugar during crop bloom times, will the bees still forage as frequently? Or is there a chance of a decrease in pollination services to the crops because of the feeding? Perhaps the authors could explore this a bit.

They will forage for pollen or nectar if they are fed syrup in the first case and protein supplement in the second case


Line 458- Is this total pollen? Why not examine crop pollen vs everything else?

All data, results and discussion about pollen weight has been removed as we now believe it was confusing and did not add anything relevant to the study.

6. PLOS authors have the option to publish the peer review history of their article ([what does this mean?](https://journals.plos.org/plosone/s/editorial-and-peer-review-process#loc-peer-review-history)). If published, this will include your full peer review and any attached files.


If you choose “no”, your identity will remain anonymous but your review may still be made public.


**Do you want your identity to be public for this peer review?** For information about this choice, including consent withdrawal, please see our [Privacy Policy](https://www.plos.org/privacy-policy).

Reviewer #1: No

Reviewer #2: No


[NOTE: If reviewer comments were submitted as an attachment file, they will be attached to this email and accessible via the submission site. Please log into your account, locate the manuscript record, and check for the action link "View Attachments". If this link does not appear, there are no attachment files to be viewed.]


While revising your submission, please upload your figure files to the Preflight Analysis and Conversion Engine (PACE) digital diagnostic tool,<https://pacev2.apexcovantage.com/>. PACE helps ensure that figures meet PLOS requirements. To use PACE, you must first register as a user. Registration is free. Then, login and navigate to the UPLOAD tab, where you will find detailed instructions on how to use the tool. If you encounter any issues or have any questions when using PACE, please email us at [figures@plos.org](mailto:figures@plos.org). Please note that Supporting Information files do not need this step.
