# Supplementary material for: The impact of lowbush blueberry (Vaccinium angustifolium Ait.) and cranberry (Vaccinium macrocarpon Ait.) pollination on honey bee (Apis mellifera L.) colony health status
Source: PLoS One. 2020 Jan 24;15(1):e0227970. doi: 10.1371/journal.pone.0227970 (PMC6980599; doi:10.1371/journal.pone.0227970)
Supplement: Peer review history

# Peer review: Decision Letter 0

**DOI:** 10.1371/journal.pone.0227970.r001

**Authors:** Manu E. Saunders

28 Aug 2019

PONE-D-19-18647

The impact of lowbush blueberry (Vaccinium angustifolium Ait.) and cranberry (Vaccinium macrocarpon Ait.) pollination on honey bee (Apis mellifera L.) colony health status

PLOS ONE

Dear Dr Giovenazzo,

Thank you for submitting your manuscript to PLOS ONE. After careful consideration, we feel that it has merit but does not fully meet PLOS ONE’s publication criteria as it currently stands. Therefore, we invite you to submit a revised version of the manuscript that addresses the points raised during the review process.

Please address the attached comments from myself and two reviewers. In particular, the manuscript requires much more detail and clarification about study methods and data analysis to enable the reader to verify the results.

We would appreciate receiving your revised manuscript by Oct 12 2019 11:59PM. When you are ready to submit your revision, log on to https://www.editorialmanager.com/pone/ and select the 'Submissions Needing Revision' folder to locate your manuscript file.

If you would like to make changes to your financial disclosure, please include your updated statement in your cover letter.

To enhance the reproducibility of your results, we recommend that if applicable you deposit your laboratory protocols in protocols.io, where a protocol can be assigned its own identifier (DOI) such that it can be cited independently in the future. For instructions see: http://journals.plos.org/plosone/s/submission-guidelines#loc-laboratory-protocols

Please include the following items when submitting your revised manuscript:

- A rebuttal letter that responds to each point raised by the academic editor and reviewer(s). This letter should be uploaded as separate file and labeled 'Response to Reviewers'.
- A marked-up copy of your manuscript that highlights changes made to the original version. This file should be uploaded as separate file and labeled 'Revised Manuscript with Track Changes'.
- An unmarked version of your revised paper without tracked changes. This file should be uploaded as separate file and labeled 'Manuscript'.

Please note while forming your response, if your article is accepted, you may have the opportunity to make the peer review history publicly available. The record will include editor decision letters (with reviews) and your responses to reviewer comments. If eligible, we will contact you to opt in or out.

We look forward to receiving your revised manuscript.

Kind regards,

Manu E. Saunders

Academic Editor

PLOS ONE

Journal Requirements:

1. When submitting your revision, we need you to address these additional requirements.

Please ensure that your manuscript meets PLOS ONE's style requirements, including those for file naming. The PLOS ONE style templates can be found at

http://www.journals.plos.org/plosone/s/file?id=wjVg/PLOSOne_formatting_sample_main_body.pdf and http://www.journals.plos.org/plosone/s/file?id=ba62/PLOSOne_formatting_sample_title_authors_affiliations.pdf

2.  Please include captions for your Supporting Information files at the end of your manuscript, and update any in-text citations to match accordingly. Please see our Supporting Information guidelines for more information: http://journals.plos.org/plosone/s/supporting-information.

3. In your Methods section, please provide additional information regarding the permits you obtained for the work. Please ensure you have included the full name of the authority that approved the field site access and, if no permits were required, a brief statement explaining why.

4. Thank you for stating the following in the Financial Disclosure section:

PG received  research grant from Bayer Crop Science. Bayer Crop Science participated in the initial study design but was not involved in the final study desing nor the data collection, data anlysis, decision to publish and preparation of manuscript.

We note that you received funding from a commercial source: Bayer Crop Science.

Please provide an amended Competing Interests Statement that explicitly states this commercial funder, along with any other relevant declarations relating to employment, consultancy, patents, products in development, marketed products, etc.

Within this Competing Interests Statement, please confirm that this does not alter your adherence to all PLOS ONE policies on sharing data and materials by including the following statement: "This does not alter our adherence to PLOS ONE policies on sharing data and materials.” (as detailed online in our guide for authors http://journals.plos.org/plosone/s/competing-interests).  If there are restrictions on sharing of data and/or materials, please state these. Please note that we cannot proceed with consideration of your article until this information has been declared.

Please include your amended Competing Interests Statement within your cover letter. We will change the online submission form on your behalf.

Please know it is PLOS ONE policy for corresponding authors to declare, on behalf of all authors, all potential competing interests for the purposes of transparency. PLOS defines a competing interest as anything that interferes with, or could reasonably be perceived as interfering with, the full and objective presentation, peer review, editorial decision-making, or publication of research or non-research articles submitted to one of the journals. Competing interests can be financial or non-financial, professional, or personal. Competing interests can arise in relationship to an organization or another person. Please follow this link to our website for more details on competing interests: http://journals.plos.org/plosone/s/competing-interests

5.  We note that Figure 1 in your submission contain map/satellite images which may be copyrighted. All PLOS content is published under the Creative Commons Attribution License (CC BY 4.0), which means that the manuscript, images, and Supporting Information files will be freely available online, and any third party is permitted to access, download, copy, distribute, and use these materials in any way, even commercially, with proper attribution. For these reasons, we cannot publish previously copyrighted maps or satellite images created using proprietary data, such as Google software (Google Maps, Street View, and Earth). For more information, see our copyright guidelines: http://journals.plos.org/plosone/s/licenses-and-copyright.

 We require you to either (1) present written permission from the copyright holder to publish these figures specifically under the CC BY 4.0 license, or (2) remove the figures from your submission:

1. You may seek permission from the original copyright holder of Figure 1 to publish the content specifically under the CC BY 4.0 license. 

 We recommend that you contact the original copyright holder with the Content Permission Form (http://journals.plos.org/plosone/s/file?id=7c09/content-permission-form.pdf) and the following text:

“I request permission for the open-access journal PLOS ONE to publish XXX under the Creative Commons Attribution License (CCAL) CC BY 4.0 (http://creativecommons.org/licenses/by/4.0/). Please be aware that this license allows unrestricted use and distribution, even commercially, by third parties. Please reply and provide explicit written permission to publish XXX under a CC BY license and complete the attached form.”

 Please upload the completed Content Permission Form or other proof of granted permissions as an "Other" file with your submission.

 In the figure caption of the copyrighted figure, please include the following text: “Reprinted from [ref] under a CC BY license, with permission from [name of publisher], original copyright [original copyright year].”

1. If you are unable to obtain permission from the original copyright holder to publish these figures under the CC BY 4.0 license or if the copyright holder’s requirements are incompatible with the CC BY 4.0 license, please either i) remove the figure or ii) supply a replacement figure that complies with the CC BY 4.0 license. Please check copyright information on all replacement figures and update the figure caption with source information. If applicable, please specify in the figure caption text when a figure is similar but not identical to the original image and is therefore for illustrative purposes only.The following resources for replacing copyrighted map figures may be helpful:

 USGS National Map Viewer (public domain): http://viewer.nationalmap.gov/viewer/

The Gateway to Astronaut Photography of Earth (public domain): http://eol.jsc.nasa.gov/sseop/clickmap/

Maps at the CIA (public domain): https://www.cia.gov/library/publications/the-world-factbook/index.html and https://www.cia.gov/library/publications/cia-maps-publications/index.html

NASA Earth Observatory (public domain): http://earthobservatory.nasa.gov/

Landsat: http://landsat.visibleearth.nasa.gov/

USGS EROS (Earth Resources Observatory and Science (EROS) Center) (public domain): http://eros.usgs.gov/#

Natural Earth (public domain): http://www.naturalearthdata.com/

Additional Editor Commens:

I agree with both reviewers that this paper is interesting but requires some major revisions to improve clarity. In particular, the claims made in the discussion may be valid, but it is hard for the reader to verify this because the methodological details are limited or confusing. See Reviewer 1's comprehensive comments about this.

The description of the study design set up is quite confusing - it would help the read if the authors could specifically describe how many colonies were under which treatment, and at what times. For example, in Table 1 the authors could include (n = ?) in each box to show how many of the 20 colonies were in each treatment at a given stage of the experiment.

In addition, the statistical analysis requires some more clarification. I also found the description of the modelling approach confusing (lines 235-259). I agree that each colony is an experimental unit, but because some colonies are moved, there is a 'time X location' interaction here - it's not clear if this was taken into consideration. Also see Reviewer 2's comment about addressing pseudoreplication. The details of the mixed models are not given - this needs to be more clear. Should the mixed models include colony number as a random effect to account for movement issue?

[Note: HTML markup is below. Please do not edit.]

Reviewers' comments:

Reviewer's Responses to Questions

**Comments to the Author**

1. Is the manuscript technically sound, and do the data support the conclusions?

The manuscript must describe a technically sound piece of scientific research with data that supports the conclusions. Experiments must have been conducted rigorously, with appropriate controls, replication, and sample sizes. The conclusions must be drawn appropriately based on the data presented. 

Reviewer #1: No

Reviewer #2: Yes

**********

2. Has the statistical analysis been performed appropriately and rigorously? 

Reviewer #1: I Don't Know

Reviewer #2: Yes

**********

3. Have the authors made all data underlying the findings in their manuscript fully available?

The PLOS Data policy requires authors to make all data underlying the findings described in their manuscript fully available without restriction, with rare exception (please refer to the Data Availability Statement in the manuscript PDF file). The data should be provided as part of the manuscript or its supporting information, or deposited to a public repository. For example, in addition to summary statistics, the data points behind means, medians and variance measures should be available. If there are restrictions on publicly sharing data—e.g. participant privacy or use of data from a third party—those must be specified.

Reviewer #1: No

Reviewer #2: Yes

**********

4. Is the manuscript presented in an intelligible fashion and written in standard English?

PLOS ONE does not copyedit accepted manuscripts, so the language in submitted articles must be clear, correct, and unambiguous. Any typographical or grammatical errors should be corrected at revision, so please note any specific errors here.

Reviewer #1: No

Reviewer #2: Yes

**********

5. Review Comments to the Author

Please use the space provided to explain your answers to the questions above. You may also include additional comments for the author, including concerns about dual publication, research ethics, or publication ethics. (Please upload your review as an attachment if it exceeds 20,000 characters)

Reviewer #1: This study aimed to determine whether using honey bee colonies for commercial pollination of cranberries, blueberries or both resulted in negative impacts on their health compared to colonies used for honey production only. They hypothesised that the bees exposed to more diverse and abundant floral resources ie the honey production colonies, would be healthier (as assessed by measures of colony development and parasite and pathogen loads) than their counterparts exposed to a reduced floral diversity (monoculture crops). Their key finding was that bees used for commercialisation of cranberry (both cranberry only and cranberry + blueberry) experiences significantly less weight gain compared to control and blueberry only bees. They also saw differences in capped brood area, virus loads and Nosema levels (blueberry only) during pollination seasons.

While I think this experimental setup is sound and the results are novel and interesting, the conclusions claim far more than can reasonably be inferred from the results, some of which do not appear to be reported at all or are there but not described in sufficient detail. Furthermore, the writing is often unclear or does not flow well.

General points:

1. While the difference in weight gain appears to be the most striking result, I believe too much is concluded from this. While this result is consistent with the capped brood data during June and July, by August and May2017 the brood area had returned to comparable levels which suggests to me the differences were largely due to honey stores which is not surprising given that the honey producing colonies were presumably put into an area with a particularly good honey flow which the authors state in the discussion was during cranberry pollination period.

2. This manuscript discussed negative effects of pollination on bee health but it seems to me that all colonies remained largely healthy. The paper discusses colony mortality but nowhere is this data shown so I think it is difficult to infer any ‘carry over’ effects when it seems all colonies made it through winter with little, if any difference to any of the metrics assessed.

3. I feel this manuscript would benefit from an overall rewrite to improve clarity and flow. See specific points for some examples.

Specific points:

L31. Sacbrood is one word

L36. You list three things here which are quite vague and overlapping without going into what you mean. You only give the example of increasing monoculture crops. It would be better if you gave further examples of what you mean, or stick to just discussing monocultures.

L46050. This paragraph doesn’t flow smoothly, there are a number of facts and numbers but reported quite differently and it is confusing to read eg blueberry fruit set rises from 30% to 100% and Carberry increases by 50% but from what to what? It reads like something is missing when you have included that info for blueberries.

L52. Add (Canada) at the first mention of Quebec

L52-58. This paragraph, along with a number of others throughout the manuscript, including the previous one, to me does not flow well and instead just reads like a list of stats. I think this could benefit from some re-wording to improve the flow.

L60. Change ‘has’ to ‘can have’ etc. Please go through this paragraph to tone down statements. I don’t think previous research has been conclusive enough that using bees for pollination of monocultures always has a detrimental effect on their health, hence why your study is interesting.

L76-77. This sentence is confusing, do you mean in the wider environment compared to the crop or generally bees need pollen and nectar? If the first is what you mean, I think this is commonly untrue, if the latter, it seems an unnecessary sentence in a strange place.

L91. Specify you mean a negative impact

L93-96. I don’t quite understand this? Do you mean this manuscript is the first part of a two-part study? This seems unusual to me but I don’t know what the journal’s guidelines are with these matters.

L107. This is the first mention of a farmland apiary and you don’t seem to use the term again. I think perhaps it could be worded differently as blueberry and cranberry farm are also farmland. Perhaps state what kind of farm it is?

L112. You don’t need ‘was carefully selected’

L123. By ‘from the dates presented earlier’ do you mean May 2016?

L124. This would read better “for blueberries (5 colonies),cranberries (5 colonies) or both (5 colonies)”

L132. Is “Propolis-etc..” the name of the manufacturer? If so please also give the location.

L140. This table would be clearer with dates across the top and location by colour, as with Table 3

L191-229. Were the different life stages analysed separately or together? How many of each life stage were analysed? How were individuals pooled? 280 individuals is a lot to be collected to only analyse 10, was there a particular reason for this? I’m not familiar with the pathogen RNA/DNA kit, does it somehow enrich your samples? Can you explain your qPCR controls/standards? How did you calculate per reaction copy number? Did using neat RNA/DNA yield quantifiable results for all samples without the need to dilute any? Etc etc ....more info is needed in this section.

L230. When were Varroa drops recorded? They aren’t mentioned in your sampling plan.

L237. I’m afraid I don’t know enough about these analyses to comment on this aspect of the manuscript.

L270. Why do you only discuss composition of the good foraging landscape at 5km radii? What about at 1km and 3km. Maybe this information should be presented in a supplementary table.

L302. Specify between all groups

L307. These dates are confusing and don’t seem to align with the sampling schedule.

L324. Please state ‘compared to control and cranberry’ and clarify that it was only at the first date of sampling in blueberry pollination, you don’t have the data to compare at the second date. Also the pollen weight did increase in the hives that were in blueberry farms from the first to second sampling dates. So perhaps more blueberry pollen was available/collected in the later part of the flowering? I think you need to be more cautious in your reporting of this data. Also, unless I am misunderstanding your data, there were other significant results eg before pollination the double ms had collected less pollen than the control. Please explain the missing data.

L345. It can also be seen that the amount of capped brood returned to comparable amounts between all treatments in August and remained the same in the following spring. You should be careful to report results in an unbiased way and ensure selectively mentioning some parts and not others isn’t leading to making unsupported conclusions.

L356. You don’t mention queen loss in the methods or give the actual results. You need to provide more information on what you saw. Grouping swarming and queen supersedure and loss all together is not necessarily appropriate as they can be due to vastly differently factors. Ie swarming can be due to a successful colony outgrowing the hive boxes, or supersedure may follow the colony’s removal of a queen that is not performing.

L361-382. This section is quite unclear to me ie:

• it mentions forager bees in particular but doesn’t report any results differentiated by caste

• If “ABPV was in one … sample only” then “there was no significant difference between groups” seems an odd sentence?

• Stating that some viruses were “rarely detected” in particular castes isn’t enough and the results should be reported, summarised in a supplementary table perhaps?

• “The most important infection” needs rewording

• Etc. Please revisit this section and clarify.

Tables 4 and 5: personally I find this information hard to read in a table and would prefer graphs or a table but with grey-scaling according to copy number. Please specify why Spring 2017 SBV data is missing.

L408. The methods stated that no differentiation was made between N. apis and N. ceranae so how do you know it is N. ceranae?

L417. Why do you multiple the spores/bee by 10^6?

L424. I would like to see the actual mite drop data in the supp.

L438-444. I believe these conclusion and confirmations of other groups’ findings to be largely overblown.eg “as long as the vegetation in the vicinity is protected and left in a natural state” wasn’t something you tested. You didn’t assess the attractiveness of particular crops/floral resources int his study.

L445-448. are unnecessary

L449-455. This seems odd her. You didn’t assess attractiveness nor pollination/crop yield.

L461. What are these observations?

L463. If you suspect the pollen traps did not work consistently for colonies in blueberries, it suggests you think the data from this point is an underestimate. If so exercise caution with your conclusion that bees were able to collect less pollen from blueberries.

L465-466. Why do you assume this?

L473-474. You cannot confirm this from your data

L481. What does “critical decline” mean in this case?

L496. Did it rain at all sites? Perhaps if the bees could not fly and forage this is the reason for the weight gain differences at this period?

L516. You have winter mortality but nowhere is this mentioned in methods or results.L529. This is misleading, some differences were seen prior to pollination too, and afterwards results differences disappeared.

L539-540. This is inaccurate, or you have not shown important results.

L547. See Welch et al 2009, Glenny et al 2017

L563-565. I think your wording of the research question needs changing

L573-576. Was this data published previously? Is there a reference?

Reviewer #2: Overall, I thought this was an interesting study and is well-written. Below are a few edits/comments that I think will potentially make the manuscript stronger.

Line 16- change to "...crops benefit from the presence of honey bees..."

Lines 66-70- I found this section a bit hard to read. I think the authors could re-word this to make it flow better.

Lines 93-96- I do not think this is needed and it should be deleted.

Line 152- change to "Data were..." Data is plural and I think the authors made this mistake a few times within the manuscript. See line 154- "...Quebec and were analysed..."

Additionally see lines 170 and 175.... Data were (or are)…

Line 245- I do not understand how changing the alpha level limits pseudoreplication. You either have or have not pseudoreplication within your study...regardless of the alpha level. Perhaps clarify this within the manuscript.

Line 357- This occurred in 9 of 20 colonies? This seems like a lot? I'm confused why you would have had numerous colonies swarm from the cranberry MS if the colonies were not obtaining enough resources and were decreasing in weight. I would assume that swarming would have been rare in this treatment?

Line 420- change to (HSD)

Line 446- I think this statement needs a citation. And by "wild" do you mean feral honey bee colonies or native bees in general?

Lines 584-587- If you feed colonies substantive quantities of pollen and/or sugar during crop bloom times, will the bees still forage as frequently? Or is there a chance of a decrease in pollination services to the crops because of the feeding? Perhaps the authors could explore this a bit.

Line 458- Is this total pollen? Why not examine crop pollen vs everything else?

**********

6. PLOS authors have the option to publish the peer review history of their article (what does this mean?). If published, this will include your full peer review and any attached files.

If you choose “no”, your identity will remain anonymous but your review may still be made public.

**Do you want your identity to be public for this peer review?** For information about this choice, including consent withdrawal, please see our Privacy Policy.

Reviewer #1: No

Reviewer #2: No

[NOTE: If reviewer comments were submitted as an attachment file, they will be attached to this email and accessible via the submission site. Please log into your account, locate the manuscript record, and check for the action link "View Attachments". If this link does not appear, there are no attachment files to be viewed.]

While revising your submission, please upload your figure files to the Preflight Analysis and Conversion Engine (PACE) digital diagnostic tool, https://pacev2.apexcovantage.com/. PACE helps ensure that figures meet PLOS requirements. To use PACE, you must first register as a user. Registration is free. Then, login and navigate to the UPLOAD tab, where you will find detailed instructions on how to use the tool. If you encounter any issues or have any questions when using PACE, please email us at figures@plos.org. Please note that Supporting Information files do not need this step.

# Author comment: Author response to Decision Letter 0

**DOI:** 10.1371/journal.pone.0227970.r002

11 Oct 2019

Dear Editor and reviewers, 

You will find detailled responses to each of your comments in the attached file named: PONE-D-19-18647_Rebuttal letter-Response to reviewers.docx.

Kind regards

Pierre Giovenazzo

Attachment Submitted filename: PONE-D-19-18647_Rebuttal letter-Response to reviewers.docx Click here for additional data file.

# Peer review: Decision Letter 1

**DOI:** 10.1371/journal.pone.0227970.r003

**Authors:** Manu E. Saunders

20 Nov 2019

PONE-D-19-18647R1

The impact of lowbush blueberry (Vaccinium angustifolium Ait.) and cranberry (Vaccinium macrocarpon Ait.) pollination on honey bee (Apis mellifera L.) colony health status

PLOS ONE

Dear Dr Giovenazzo,

Thank you for submitting your manuscript to PLOS ONE. After careful consideration, we feel that it has merit but does not fully meet PLOS ONE’s publication criteria as it currently stands. Therefore, we invite you to submit a revised version of the manuscript that addresses the points raised during the review process.

Thank you for revising the manuscript. I think there are still some issues with the clarity around some methodological issues. Please address the comments from Reviewer 1, particularly regarding rain data and no long term effect of viruses.

I have also provide some comments below that need to be addressed.

We would appreciate receiving your revised manuscript by Jan 04 2020 11:59PM. When you are ready to submit your revision, log on to https://www.editorialmanager.com/pone/ and select the 'Submissions Needing Revision' folder to locate your manuscript file.

If you would like to make changes to your financial disclosure, please include your updated statement in your cover letter.

To enhance the reproducibility of your results, we recommend that if applicable you deposit your laboratory protocols in protocols.io, where a protocol can be assigned its own identifier (DOI) such that it can be cited independently in the future. For instructions see: http://journals.plos.org/plosone/s/submission-guidelines#loc-laboratory-protocols

Please include the following items when submitting your revised manuscript:

- A rebuttal letter that responds to each point raised by the academic editor and reviewer(s). This letter should be uploaded as separate file and labeled 'Response to Reviewers'.
- A marked-up copy of your manuscript that highlights changes made to the original version. This file should be uploaded as separate file and labeled 'Revised Manuscript with Track Changes'.
- An unmarked version of your revised paper without tracked changes. This file should be uploaded as separate file and labeled 'Manuscript'.

Please note while forming your response, if your article is accepted, you may have the opportunity to make the peer review history publicly available. The record will include editor decision letters (with reviews) and your responses to reviewer comments. If eligible, we will contact you to opt in or out.

We look forward to receiving your revised manuscript.

Kind regards,

Manu E. Saunders

Academic Editor

PLOS ONE

Additional Editor Comments (if provided):

(Manu Saunders)

Please include the sample size in the Abstract.

Throughout the manuscript, you discuss the effect of pollination services in each crop as being positive or negative on the bees – for example, line 34, line 469 – 475, and elsewhere. The language used implies that the crop itself is having the negative effect on colony health. However, the landscape effect is confounded with the crop type on each farm – and there is only one site for each treatment. This needs to be very clear throughout. You did not test the quality of each crop’s floral resources, so I think it’s important to clarify that you are talking about the effect of each study site, not cranberry or blueberry pollination generally. For example, you may find very different effects in a more diverse cranberry field in another landscape context. I imagine the cranberry effect will also depend on the type of enterprise – was this a natural wetland system, or a constructed commercial system? This will of course influence the type of vegetation around the farm and is unlikely to be representative of cranberry farms everywhere. Please add more detail where necessary and clarify this in the discussion.

Line 267-269: I don’t think the previous reviewer’s comment about inference and p values has been addressed appropriately. Fixing the p value threshold at 0.01 does not ‘account for pseudoreplication’. Also, p values are often of limited use with very small sample sizes, as is the case here (n = 5 for each treatment). I suggest rephrasing this sentence something like “We used a p value threshold of 0.01. However, we caution interpretation of significance in our results because of the small sample size and presence of pseudoreplication.”

[Note: HTML markup is below. Please do not edit.]

Reviewers' comments:

Reviewer's Responses to Questions

**Comments to the Author**

1. If the authors have adequately addressed your comments raised in a previous round of review and you feel that this manuscript is now acceptable for publication, you may indicate that here to bypass the “Comments to the Author” section, enter your conflict of interest statement in the “Confidential to Editor” section, and submit your "Accept" recommendation.

Reviewer #1: (No Response)

**********

2. Is the manuscript technically sound, and do the data support the conclusions?

The manuscript must describe a technically sound piece of scientific research with data that supports the conclusions. Experiments must have been conducted rigorously, with appropriate controls, replication, and sample sizes. The conclusions must be drawn appropriately based on the data presented. 

Reviewer #1: Yes

**********

3. Has the statistical analysis been performed appropriately and rigorously? 

Reviewer #1: I Don't Know

**********

4. Have the authors made all data underlying the findings in their manuscript fully available?

The PLOS Data policy requires authors to make all data underlying the findings described in their manuscript fully available without restriction, with rare exception (please refer to the Data Availability Statement in the manuscript PDF file). The data should be provided as part of the manuscript or its supporting information, or deposited to a public repository. For example, in addition to summary statistics, the data points behind means, medians and variance measures should be available. If there are restrictions on publicly sharing data—e.g. participant privacy or use of data from a third party—those must be specified.

Reviewer #1: Yes

**********

5. Is the manuscript presented in an intelligible fashion and written in standard English?

PLOS ONE does not copyedit accepted manuscripts, so the language in submitted articles must be clear, correct, and unambiguous. Any typographical or grammatical errors should be corrected at revision, so please note any specific errors here.

Reviewer #1: Yes

**********

6. Review Comments to the Author

Please use the space provided to explain your answers to the questions above. You may also include additional comments for the author, including concerns about dual publication, research ethics, or publication ethics. (Please upload your review as an attachment if it exceeds 20,000 characters)

Reviewer #1: I thank the authors for taking on board my comments and I feel the manuscript is now vastly improved. I think that making the hive weight data more central and removing the pollen stores data have made the message clearer and the manuscript stronger. The additional information and rewording of many parts of the methods and results mean the revised manuscript is much clearer and easier to follow. That being said, I have a few minor points that I would like to see addressed before the manuscript is accepted.

The addition of the rain data I think is important as it seems to me this could played a major role in how the data turned out. It seems to me significant that during cranberry pollination it rained on half the days (14/28), compared to more like one day in five in the farmland (5/28). This could potentially explain a large part of your data (both due to diminished nectar production, as you point out, and also in days/hours the bees could actually go out and forage). Whilst you have discussed this to a point, I think this important caveat should be included in the summary part of the discussion.

Largely, where differences are seen, eg in Nosema levels, and in SBV and BQCV virulence, they seem to return to ‘normal’ by the following spring eg no long term effect. Whilst an effect in one year is important and less healthy hives going into winter isn’t good, I think at some point in your discussion you should state that mostly the hives (in this year at least) were able to return to similar ‘health’ as their control counterparts.

L 426 – I would prefer it if you stated that the Nosema levels had returned to no significant differences by the flowing spring

L544 – relationship not relation

L555 – I think Globally is too strong a word here, something like overall would be better

**********

7. PLOS authors have the option to publish the peer review history of their article (what does this mean?). If published, this will include your full peer review and any attached files.

If you choose “no”, your identity will remain anonymous but your review may still be made public.

**Do you want your identity to be public for this peer review?** For information about this choice, including consent withdrawal, please see our Privacy Policy.

Reviewer #1: No

[NOTE: If reviewer comments were submitted as an attachment file, they will be attached to this email and accessible via the submission site. Please log into your account, locate the manuscript record, and check for the action link "View Attachments". If this link does not appear, there are no attachment files to be viewed.]

While revising your submission, please upload your figure files to the Preflight Analysis and Conversion Engine (PACE) digital diagnostic tool, https://pacev2.apexcovantage.com/. PACE helps ensure that figures meet PLOS requirements. To use PACE, you must first register as a user. Registration is free. Then, login and navigate to the UPLOAD tab, where you will find detailed instructions on how to use the tool. If you encounter any issues or have any questions when using PACE, please email us at figures@plos.org. Please note that Supporting Information files do not need this step.

# Author comment: Author response to Decision Letter 1

**DOI:** 10.1371/journal.pone.0227970.r004

19 Dec 2019

PONE-D-19-18647R1

The impact of lowbush blueberry (Vaccinium angustifolium Ait.) and cranberry (Vaccinium macrocarpon Ait.) pollination on honey bee (Apis mellifera L.) colony health status

PLOS ONE

Editor Manu Saunders

Please include the sample size in the Abstract.

We added it (line 23).

Throughout the manuscript, you discuss the effect of pollination services in each crop as being positive or negative on the bees – for example, line 34, line 469 – 475, and elsewhere. The language used implies that the crop itself is having the negative effect on colony health. However, the landscape effect is confounded with the crop type on each farm – and there is only one site for each treatment. This needs to be very clear throughout. You did not test the quality of each crop’s floral resources, so I think it’s important to clarify that you are talking about the effect of each study site, not cranberry or blueberry pollination generally. For example, you may find very different effects in a more diverse cranberry field in another landscape context. I imagine the cranberry effect will also depend on the type of enterprise – was this a natural wetland system, or a constructed commercial system? This will of course influence the type of vegetation around the farm and is unlikely to be representative of cranberry farms everywhere. Please add more detail where necessary and clarify this in the discussion.

We agree and we rephrased sentences accordingly (lines 32, 33, 483, 494, 506, 516, 608-609).

Line 267-269: I don’t think the previous reviewer’s comment about inference and p values has been addressed appropriately. Fixing the p value threshold at 0.01 does not ‘account for pseudoreplication’. Also, p values are often of limited use with very small sample sizes, as is the case here (n = 5 for each treatment). I suggest rephrasing this sentence something like “We used a p value threshold of 0.01. However, we caution interpretation of significance in our results because of the small sample size and presence of pseudoreplication.”

We agree as this is exactly what we intended to explain. We added this clarification (lines 281-282).

Reviewer #1: 

I thank the authors for taking on board my comments and I feel the manuscript is now vastly improved. I think that making the hive weight data more central and removing the pollen stores data have made the message clearer and the manuscript stronger. The additional information and rewording of many parts of the methods and results mean the revised manuscript is much clearer and easier to follow. That being said, I have a few minor points that I would like to see addressed before the manuscript is accepted.

The addition of the rain data I think is important as it seems to me this could played a major role in how the data turned out. It seems to me significant that during cranberry pollination it rained on half the days (14/28), compared to more like one day in five in the farmland (5/28). This could potentially explain a large part of your data (both due to diminished nectar production, as you point out, and also in days/hours the bees could actually go out and forage). Whilst you have discussed this to a point, I think this important caveat should be included in the summary part of the discussion.

We agree. We added a "Precipitation" paragraph for each of the main sections: Material and methods (lines 171-176), Results (lines 348-354) and Discussion (lines 495-502).

Largely, where differences are seen, eg in Nosema levels, and in SBV and BQCV virulence, they seem to return to ‘normal’ by the following spring eg no long term effect. Whilst an effect in one year is important and less healthy hives going into winter isn’t good, I think at some point in your discussion you should state that mostly the hives (in this year at least) were able to return to similar ‘health’ as their control counterparts.

We agree and added two sentences accordingly: Lines 577-580 and lines 608-609.

L 426 – I would prefer it if you stated that the Nosema levels had returned to no significant differences by the flowing spring

We agree and added the sentence (lines 454-455)

L544 – relationship not relation

We corrected it (line 586).

L555 – I think Globally is too strong a word here, something like overall would be better

We corrected it (line 599).

# Editor report: Decision Letter 2

**DOI:** 10.1371/journal.pone.0227970.r005

**Authors:** Manu E. Saunders

6 Jan 2020

The impact of lowbush blueberry (Vaccinium angustifolium Ait.) and cranberry (Vaccinium macrocarpon Ait.) pollination on honey bee (Apis mellifera L.) colony health status

PONE-D-19-18647R2

Dear Dr. Giovenazzo,

We are pleased to inform you that your manuscript has been judged scientifically suitable for publication and will be formally accepted for publication once it complies with all outstanding technical requirements.

Within one week, you will receive an e-mail containing information on the amendments required prior to publication. When all required modifications have been addressed, you will receive a formal acceptance letter and your manuscript will proceed to our production department and be scheduled for publication.

Shortly after the formal acceptance letter is sent, an invoice for payment will follow. To ensure an efficient production and billing process, please log into Editorial Manager at https://www.editorialmanager.com/pone/, click the "Update My Information" link at the top of the page, and update your user information. If you have any billing related questions, please contact our Author Billing department directly at authorbilling@plos.org.

If your institution or institutions have a press office, please notify them about your upcoming paper to enable them to help maximize its impact. If they will be preparing press materials for this manuscript, you must inform our press team as soon as possible and no later than 48 hours after receiving the formal acceptance. Your manuscript will remain under strict press embargo until 2 pm Eastern Time on the date of publication. For more information, please contact onepress@plos.org.

With kind regards,

Manu E. Saunders

Academic Editor

PLOS ONE

Additional Editor Comments (optional):

Reviewers' comments:

# Editor report: Acceptance letter

**DOI:** 10.1371/journal.pone.0227970.r006

**Authors:** Manu E. Saunders

16 Jan 2020

PONE-D-19-18647R2 

The impact of lowbush blueberry (Vaccinium angustifolium Ait.) and cranberry (Vaccinium macrocarpon Ait.) pollination on honey bee (Apis mellifera L.) colony health status 

Dear Dr. Giovenazzo:

I am pleased to inform you that your manuscript has been deemed suitable for publication in PLOS ONE. Congratulations! Your manuscript is now with our production department. 

If your institution or institutions have a press office, please notify them about your upcoming paper at this point, to enable them to help maximize its impact. If they will be preparing press materials for this manuscript, please inform our press team within the next 48 hours. Your manuscript will remain under strict press embargo until 2 pm Eastern Time on the date of publication. For more information please contact onepress@plos.org.

For any other questions or concerns, please email plosone@plos.org. 

Thank you for submitting your work to PLOS ONE.

With kind regards,

PLOS ONE Editorial Office Staff

on behalf of

Dr. Manu E. Saunders 

Academic Editor

PLOS ONE
